# Free-Base Corrole Anion

**DOI:** 10.1021/acs.joc.3c01125

**Published:** 2023-08-30

**Authors:** Arup Tarai, Jyotiprakash Mallick, Pranjali Singh, Jeanet Conradie, Sanjib Kar, Abhik Ghosh

**Affiliations:** † School of Chemical Sciences, National Institute of Science Education and Research (NISER), Bhubaneswar 752050, India; ‡ Homi Bhabha National Institute, Training School Complex, Anushakti Nagar, Mumbai 400 094, India; § Department of Chemistry, University of the Free State, P.O. Box 339, Bloemfontein 9300, Republic of South Africa; ∥ Department of Chemistry, UiT − The Arctic University of Norway, N-9037 Tromsø, Norway

## Abstract

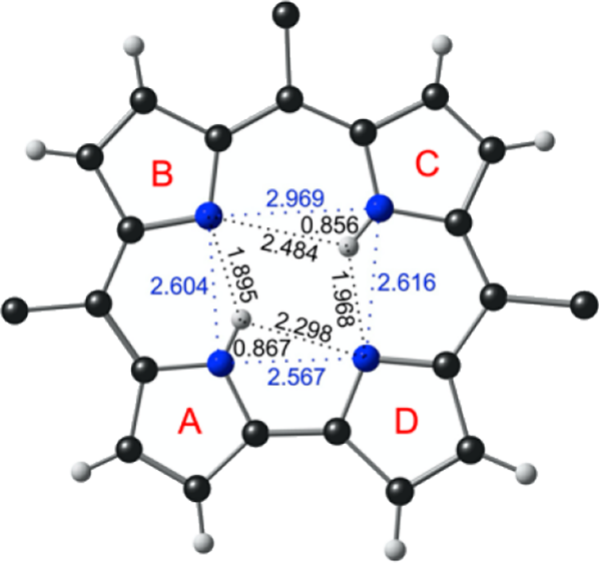

Free-base corroles have long been known to be acidic, readily undergoing deprotonation by mild bases and in polar solvents. The conjugate base, however, has not been structurally characterized until now. Presented here is a first crystal structure of a free-base corrole anion, derived from tris(*p*-cyanophenyl)corrole, as the tetrabuylammonium salt. The low-temperature (100 K) structure reveals localized hydrogens on a pair of opposite pyrrole nitrogens. DFT calculations identify such a structure as the global minimum but also point to two *cis* tautomers only 4–7 kcal/mol above the ground state. In terms of free energy, however, the *cis* tautomers are above or essentially flush with the *trans*-to-*cis* barrier so the cis tautomers are unlikely to exist or be observed as true intermediates. Thus, the hydrogen bond within each dipyrrin unit on either side of the molecular pseudo-*C*_2_ axis through C_10_ (i.e., between pyrrole rings A and B or between C and D) qualifies as or closely approaches a low-barrier hydrogen bond. Proton migration across the pseudo-*C*_2_ axis entails much higher activation energies >20 kcal/mol, reflecting the relative rigidity of the molecule along the C_1_-C_19_ pyrrole-pyrrole linkage.

## Introduction

The tautomeric structures of free-base porphyrin-type compounds reflect a fascinating interplay of hydrogen bonding, aromaticity, steric effects, and solvation.^1^ Tautomerism is also critical to applications of the compounds as components of emerging molecular-electronic and memory-storage devices.^2,3^ A great deal of study, accordingly, has been devoted to the study of tautomerism in porphyrins,^4−14^*N*-confused porphyrins,^15−18^ porphycenes,^19−24^ hydroporphyrins,^25−35^ phthalocyanines,^36−39^ expanded porphyrins,^40,41^ and corroles.^42−47^ Even when presenting the very first synthesis of a corrole, Johnson and Kay noted that it forms a stable anion.^48^ Subsequently, several researchers observed deprotonation of corroles by mild bases and even simply by polar solvents such as DMF,^49−52^ while Gross *et al*. determined the apparent acidity constant of an amphiphilic corrole.^53^ Corrole anions were found to exhibit more intense Q bands relative to the neutral, free-base forms.^54^ Kadish and coworkers found that corrole anions (H_2_Cor^–^) undergo electrooxidation and electroreduction to yield neutral radicals (H_2_Cor^•^) and anion-diradicals (H_2_Cor^•2–^), respectively.^55−57^

**Scheme 1:**
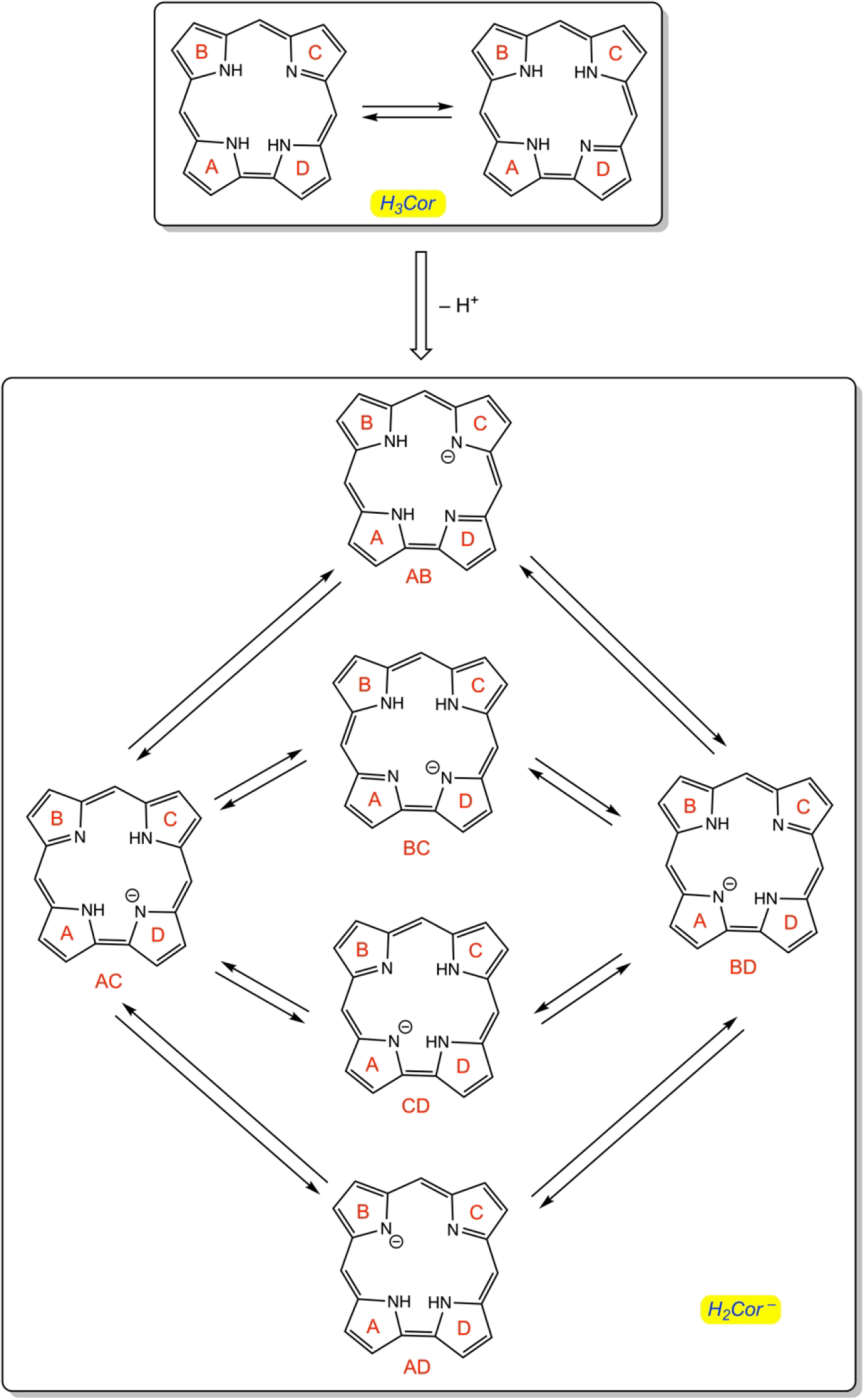
Tautomers of Free-Base Corrole and Corrole Anion The pyrrole rings are designated A–D. The corrole anion tautomers are designated by a two-letter symbol such as AC, which indicates that the NH protons reside on rings A and C. *Meso*-aryl groups have been omitted for clarity. In the present study, the *meso* aryl groups do not exert a significant impact on the relative energetics of the tautomers; thus, we shall see that *trans* tautomers AC and BD (or for that matter *cis* tautomers AB and CD) are essentially equienergetic

A key aspect of free-base corrole anions, however, has remained unclear: their structure (Scheme 1). While there is extensive evidence that free-base corroles exist as a pair of essentially equienergetic tautomers,^58−61^ key questions remain about the tautomeric structure of the anions. Is the *trans* tautomer, the one experimentally observed in this study, the only low-energy tautomer? Do the two equienergetic *trans* tautomers (AC and BD in Scheme 1) readily interconvert? If so, are *cis* tautomers (one or more among AB, CD, BC, and AD) involved as intermediates? Might the *cis* tautomers prove observable or even isolable? Herein, we present the X-ray structure of free-base tris(*p*-cyanophenyl)corrole anion and a set of DFT calculations that shed light on the these questions.

## Results and Discussion

### Synthesis and Structural Characterization

**Figure 1:**
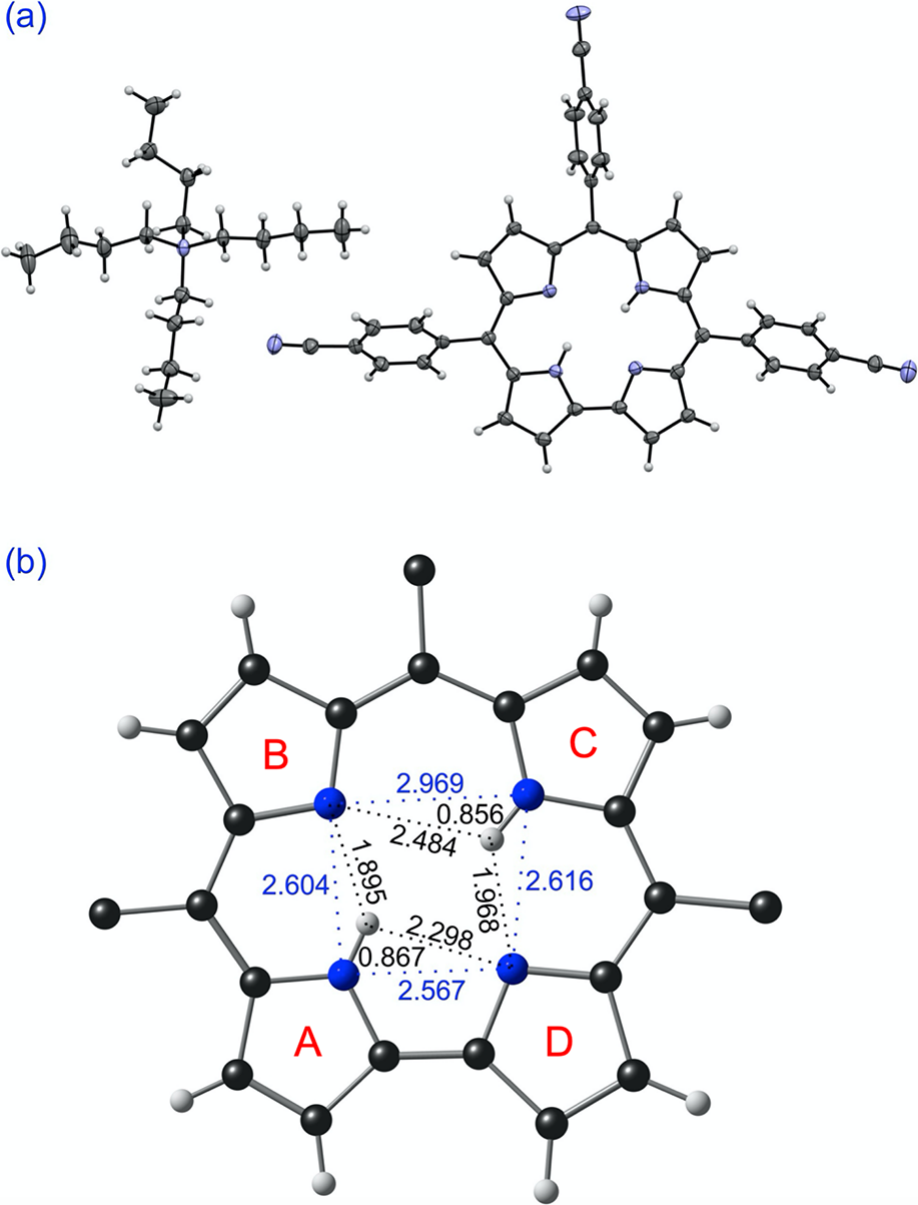
Crystal structure of TBA[H_2_Cor]: (a) thermal ellipsoid plot at 50% probability; (b) selected distances (Å) in the corrole core.

Free-base 5,10,15-tris(*p*-cyanophenyl)corrole (also, for simplicity, abbreviated as H_3_Cor) was synthesized via the water–methanol method reported by Gryko and Koszarna,^62^ with all spectroscopic data matching those reported earlier.^63^ The free-base corrole anion, H_2_Cor^–^, was obtained as the tetrabutylammonium salt, TBA[H_2_Cor], in ∼50% yield by treating the neutral free base with tetrabutylammonium fluoride (TBAF) in dichloromethane followed by crystallization from a mixture of dichloromethane and hexane. The salt was fully characterized by standard spectroscopic techniques, including UV–vis absorption, fluorescence, FT-IR, and ^1^H NMR spectroscopies, ESI mass spectrometry, and single-crystal X-ray diffraction analysis (see the Supporting Information). Deep blue crystals of TBA[H_2_Cor] were isolated from a solution of the free-base corrole and TBAF in CH_2_Cl_2_-hexane mixtures upon slow evaporation at room temperature. The crystal structure (Figure 1 and Table S1) reveals several notable features: (a) First, the corrole core is almost strictly planar, unlike that of a neutral corrole, which is invariably strongly puckered as a result of steric repulsion among the three central NH hydrogens. (b) Second, the four central nitrogens are arranged in the form of an isosceles trapezoid in which the longest side (∼3.0 Å) is antipodal to the direct pyrrole-pyrrole C_α_–C_*α*_ linkage, while the other three sides are each about 2.6 Å. (c) Third, an ordered *trans* arrangement of the central NH hydrogens is observed, with each NH hydrogen within hydrogen-bonding distance of two neighboring unprotonated nitrogens. Note that the apparently short N–H bond distances ∼0.9 Å merely reflect the fact that X-ray diffraction measures electron density, not nuclear position, which, for hydrogen, is a key distinction. It is clear nonetheless that each NH group engages in two hydrogen bonds of different lengths, a point that we will revisit below with DFT calculations. (d) Finally, the crystal structure reveals supramolecular chains of H_2_Cor units held together by C–H···N interactions involving the *p*-cyanophenyl groups. Locally, the C–H···N interactions form R22(10) and R21(5) rings, to use Etter’s terminology.^64^ Such a structure results in voids, which are occupied by tetrabutylammonium cations (see the Supporting Information for details).

### DFT Calculations

**Figure 2:**
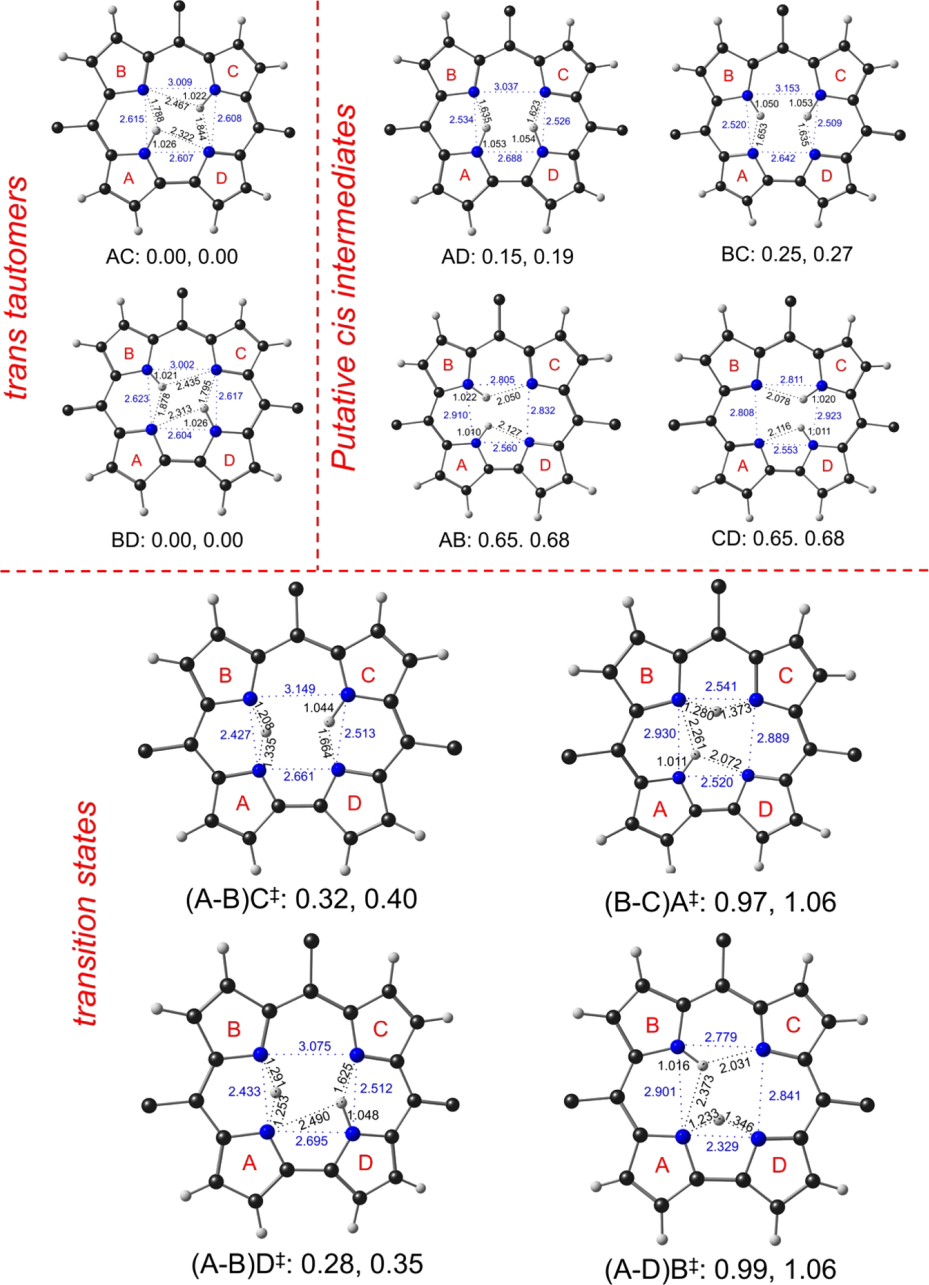
OLYP-D3 and B3LYP-D3 electronic energies (eV, in order) of *trans*-to-*cis* transition states and *cis* intermediates of H_2_Cor^–^. Selected N-H (black) and N···N (blue) distances (Å) are shown. A notation such as (A-B)C^‡^ is used for transition states, which indicates that a proton is migrating between pyrrole rings A and B, while pyrrole ring C carries a localized proton.

**Figure 3:**
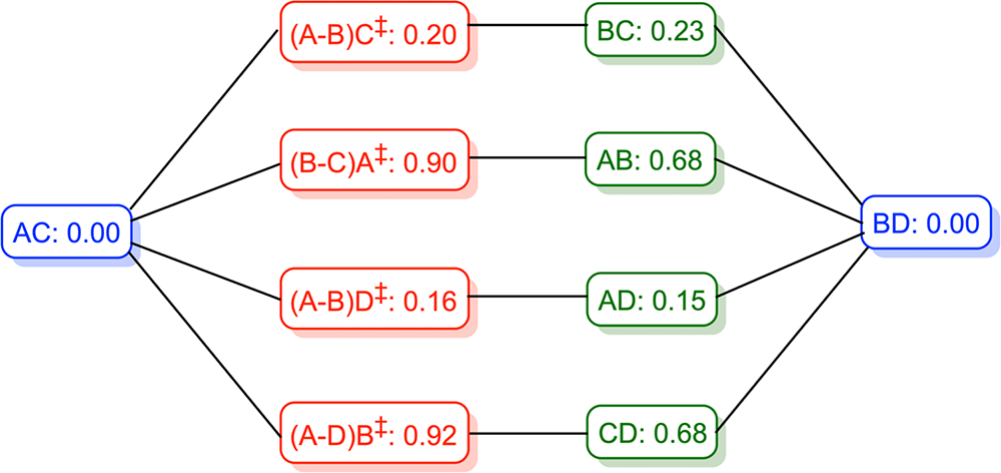
OLYP-D3 free energies (eV) of key stationary points on the potential energy surface of H_2_Cor^–^. Results for *trans* minima, *cis* intermediates, and transition states are indicated in blue, green, and red, respectively.

DFT (OLYP-D3 and B3LYP-D3) calculations with large all-electron STO-TZ2P basis sets afforded a great deal of additional insight into the structure and potential energy surface of H_2_Cor^–^ (Figures 2 and 3 and Table 1). Consistent with the crystal structure (Figure 1), the ground state corresponds to either AC or BD, i.e., the protons are attached to either pyrrole rings A and C or pyrrole rings B and D. The two tautomers are not identical, as a result of the asymmetry created by the *meso*-phenyl groups, but are equienergetic for all practical purposes. Both tautomers exhibit bifurcated hydrogen bonding, where each central NH interacts with two neighboring unprotonated nitrogens, much as in a free-base porphyrin. Unlike in porphyrin, however, the bifurcated hydrogen bonding here is *asymmetric*, with each NH group interacting via an ultrashort hydrogen bond (∼1.8 Å) and a somewhat longer hydrogen bond (∼2.4 Å). The shorter hydrogen bonds always involve a pair of pyrrole rings on one side of the molecular pseudo-*C_2_* axis (such as pyrroles A and B or C and D), whereas the longer hydrogen bonds span a pair of pyrrole rings on opposite sides of the pseudo-*C_2_* axis (pyrroles A and D or pyrrole B and C).

**Table 1:** Energetics (eV) of Selected Stationary Points for H_2_Cor^–^a

species	Δ*E* (OLYP-D3)	Δ*G* (OLYP-D3) (ν/*i*)	Δ*E* (B3LYP-D3)
BD	0.00	0.00	0.00
AC	0.00	0.00	0.01
AD	0.15	0.15	0.19
BC	0.25	0.23	0.27
AB	0.65	0.68	0.68
CD	0.65	0.68	0.68
(A-B)C^‡^	0.32	0.20 (−1074.1)	0.40
(B-C)A^‡^	0.97	0.90 (−1558.8)	1.06
(A-B)D^‡^	0.28	0.16 (−1161.9)	0.35
(A-D)B^‡^	0.99	0.92 (−1351.2)	1.06

a OLYP-D3 imaginary frequencies (cm^–1^) are indicated for transition states.

Figure 2 presents DFT geometrical highlights and energies of the tautomers and *trans*-to-*cis* transition states of H_2_Cor^–^. In terms of electronic energy, the *cis* tautomers BC and AD are remarkably stable, about 0.15–0.25 eV (3.5–6.0 kcal/mol) above the ground state (Figure 2). Their stability undoubtedly reflects the fact that they both preserve the ultrashort hydrogen bonds between pyrroles A and B and between pyrroles C and D. The low energies of the *cis* tautomers are somewhat reminiscent of isobacteriochlorin, for which the *trans* and a *cis* tautomer are essentially equienergetic and typically found to coexist.^24,27,30^ In contrast, the *cis* tautomer of a simple porphyrin is about 7–8 kcal/mol above the *trans* ground state.^9^ (It may be mentioned in passing that substituent and environmental effects may result in the stabilization and isolation of a *cis* tautomer for certain porphyrins.^65−69^) In contrast, *cis* tautomers AB and CD exhibit much higher energies, about 2/3 of an eV (16 kcal/mol) relative to the *trans* ground state, reflecting loss of preferred hydrogen bond pathways.

Proton migration appears to be most favorable along the ultrashort hydrogen bonds, i.e., from pyrrole A to B, or vice versa, or from pyrrole C to D, and vice versa. Exceedingly low activation energies (electronic energies) are associated with these migrations, only about a quarter to a third of an eV (6.5–7.5 kcal/mol) relative to the *trans* ground state. Again, for comparison, the activation barrier for proton migration is ∼12 kcal/mol for the conjugate base of free-base porphine and ∼16 kcal/mol for neutral free-base porphine. In contrast, much higher activation energies, on the order of an eV (23 kcal/mol), are associated with proton migration between rings A and D and between rings B and C of H_2_Cor^–^.

Free energy calculations add an interesting twist to the above picture (Figure 3 and Table 1). As it happens, the free energy of the transition state (A-B)C^‡^ is lower than that of the *cis* intermediate BC (see Figure 2 for an explanation of our notation for transition states). In other words, the lowest vibrational state of the *cis* intermediate is energetically higher than the transition states separating it from the *trans* tautomers. A similar scenario also applies to the *cis* intermediate AD whose free energy is almost flush with that of transition state A(C-D)^‡^. Our calculations thus suggest that the *cis* tautomers are unlikely to be observable as true intermediates. Instead, they are a part of the energy barrier through which one *trans* form tunnels to the other. In this respect, free-base corrole anion differs from both free-base porphyrin, for which the *cis* tautomer is a true intermediate on the potential energy surface,^9,12−14^ and free-base isobacteriochlorin, for which the *trans* and one *cis* tautomer coexist in equilibrium.^24,27,30^

In the absence of a free energy barrier for *trans*-to-*cis* tautomerization, the hydrogen bonds in H_2_Cor^–^ appear to satisfy the criteria for (or very closely approach) a true low-barrier hydrogen bond.^70−73^ Such hydrogen bonds have been invoked (albeit controversially^74−76^) as responsible for reaction rate enhancements in enzymatic catalysis. Recently, low-barrier hydrogen bonds have also been implicated in enzyme cooperativity.^77^ Against this backdrop, the free-base corrole anion may serve as a well-characterized and readily accessible model system for low-barrier N–H···N hydrogen bonds (several examples are known involving oxygen^78−80^). Additional spectroscopic studies are clearly warranted, of which NMR spectroscopy and nitrogen 1s X-ray photoelectron spectroscopy seem particularly promising. The latter technique has shed substantial light on the charge asymmetry of low-barrier hydrogen bonds in porphycenes.^20^

### Photophysical Properties

**Figure 4:**
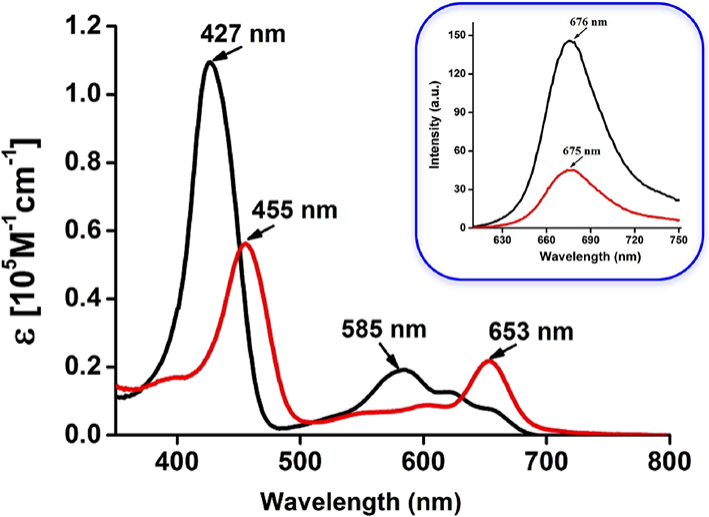
UV–Vis spectra of H_3_Cor (black) and TBA[H_2_Cor] (red) in dichloromethane. Inset: corresponding emission spectra.

Figure 4 depicts the UV–vis–NIR and fluorescence spectra of free-base H_3_Cor and TBA[H_2_Cor] in CH_2_Cl_2_, with key numerical data summarized in Table 2. Like other free-base corroles, H_3_Cor exhibits a strong Soret band at ∼427 nm and three weaker Q bands at 585, 620, and 655 nm. In contrast, TBA[H_2_Cor] exhibits a red-shifted Soret band at 455 nm, along with Q bands at 554, 600, and 653 nm. Upon excitation at 430 nm, the neutral free base exhibits strong emission with a maximum at 676 nm, a quantum yield of 6.5% (referenced to coumarin^81^), and a fluorescence lifetime of 3.79 ns. The H_2_Cor^–^ anion is also emissive, with a maximum at 675 nm, but with a much lower quantum yield of 3.3% and somewhat lower fluorescence lifetime of 3.03 ns.

**Table 2:** Photophysical Data of H_3_Cor and TBA[H_2_Cor] in CH_2_Cl_2_ at 298 K

	λ_abs_/nm (ε/10^5^ M^–1^ cm^–1^)	λ_em_ (nm)	Φ_em_^a^	τ (ns)^b^
H_3_Cor	427 (1.1), 585 (0.2), 620 (0.14), 655 (0.08)	676	0.065	3.79
TBA[H_2_Cor]	455 (0.57), 554 (0.08), 600 (0.09), 653 (0.22)	675	0.033	3.03

a Emission quantum yields were calculated using coumarin (Φ_em_ = 0.54) as the reference in degassed CH_2_Cl_2_.^80^

b Emission lifetimes were measured in degassed CH_2_Cl_2_ solution.

## Conclusions

In summary, we have presented the first crystal structure of a free-base corrole anion. The structure exhibits localized NH hydrogens on a pair of opposite pyrrole rings, which we may designate as rings A and C or equivalently as B and D. A key feature of the structure is the short-strong N–H···N hydrogen bond within each dipyrrin moiety on either side of the molecular pseudo-*C*_2_ axis through the C_10_*meso*-carbon. DFT calculations identify AC and BD as equienergetic global minima with the two *cis* tautomers AD and BC only 4–7 kcal/mol above the global minima (in terms of electronic energy). In terms of free energy, however, the *cis* tautomers are just above or essentially flush with the *trans*-to-*cis* barrier heights. Accordingly, the hydrogen bonds in free-base corrole anion qualify as or closely approach low-barrier hydrogen bonds. Proton migration across the molecular pseudo-*C*_2_ axis, on the other hand, entails much higher activation energies of >20 kcal/mol. Given the overall mobility of the low-barrier hydrogen bond system, we find it gratifying that our low-temperature crystal structure has revealed a single, ordered tautomer.

## Experimental Section

### General

Unless otherwise mentioned, all chemicals were obtained from Merck. For spectroscopic studies, HPLC-grade solvents were used. Free-base 5,10,15-tris(4-cyanophenyl)corrole was prepared as previously reported.^61^ Elemental analyses were carried out with a Euro EA elemental analyzer. UV–vis absorption spectra were acquired on a Perkin-Elmer LAMBDA-750 spectrophotometer. Emission spectra were recorded on an Edinburgh FLS920 spectrofluorimeter equipped with a Ge-detector using an optical cell with 1 cm path length. FT-IR spectra were recorded on a Perkin-Elmer spectrophotometer with samples prepared as KBr pellets. NMR spectra were obtained on a Bruker 400 MHz NMR spectrometer. Chemical shifts are expressed in parts per million (ppm) relative to residual acetonitrile (δ = 1.93). Electrospray mass spectra were recorded on a Bruker Micro TOF–QII mass spectrometer.

### Synthesis of TBA[H_2_Cor]

To a solution of free-base tris(*p*-cyanophenyl)corrole (50 mg, 0.08 mmol) in dichloromethane (25 mL), an excess of tetrabutylammonium fluoride (0.40 mmol) was added. After 10 min of continuous stirring, the reaction mixture was filtered. To the filtrate, hexane (10 mL) was added to facilitate crystallization. After 2–3 days at room temperature, deep blue block-shaped crystals of **TBA[H_2_Cor]** were isolated by filtration. Yield: 50% (37 mg). λ_max_ (nm^–1^, ε/M^–1^ cm^–1^) in CH_2_Cl_2_: 455 (5.7 x 10^4^), 554 (7.6 x 10^3^), 600 (9.2 × 10^3^), 653 (2.26 × 10^4^). ^1^H NMR (400 MHz, chloroform-*d*) δ: 8.98 (*d*, *J* = 4 Hz, 2H), 8.74 (*d*, *J* = 4.5 Hz, 2H), 8.64 (*d*, *J* = 4 Hz, 2H), 8.43 (*d*, *J* = 4.6 Hz, 2H), 8.40 (d, *J* = 8.2 Hz, 4H), 8.26 (d, *J* = 8.2 Hz, 2H), 8.03 (d, *J* = 8.3 Hz, 4H), 7.98 (d, *J* = 8.1 Hz, 2H), 2.17–2.15 (m, 8H), 0.94–0.87 (m, 8H), 0.86–0.77 (m, 8H), 0.74–0.71 (m, 12H) (Figure S6). ^13^C{^1^H} NMR (101 MHz, CDCl_3_) δ: 150.1, 147.4, 140.6, 140.4, 136.2, 135.4, 132.7, 132.0, 131.1, 130.3, 125.3, 125.0, 122.0, 119.8, 118.7, 115.8, 112.1, 109.8, 109.2, 106.9, 57.1, 23.0, 19.0, 13.4. HRMS (ESI) *m/z*: [**TBA**]^+^ Calcd for C_16_H_36_N 242.2848; Found 242.2767 and *m/z*: [**H_3_Cor** + H]^+^ Calcd for C_40_H_24_N_7_ 602.2093; Found 602.2531 (Figure S27).

### Crystal Structure Determination

Single crystals of **TBA[H_2_Cor]** were grown from the solution of **TBA[H_2_Cor]** in dichloromethane layered over hexane followed by slow evaporation under atmospheric conditions. X-ray crystallographic data on TBA[H_2_Cor] were collected on a Rigaku Oxford diffractometer (Cu Kα radiation) at 100 K, with all key parameters listed in Table S1. The data were corrected for Lorentz polarization and absorption effects. The structure was solved with intrinsic phasing methods (SHELXT40^82^) and refined by full matrix least squares on F^2^ (SHELXL-201841^83^). Hydrogen atoms were included in the refinement as riding atoms. Contributions of H atoms for the water molecules were included but were not fixed. Disordered solvent molecules were taken out using the SQUEEZE command in PLATON.^84^ The corresponding CCDC no of TBA^+^[H_2_Cor^–^] is 2258522.

## Data Availability

The data underlying this study are available in the published article and its Supporting Information.

## Supporting Information Available

Spectroscopic and crystallographic data; DFT optimized coordinates (PDF)

## References

[ref1] Stepień M.; Latos-Grazyński L. Aromaticity and Tautomerism in Porphyrins and Porphyrinoids. In Top. Heterocycl. Chem*.* 2008, Springer, Berlin, Heidelberg, 10.1007/7081_2008_4.

[ref2] Jurow M.; Schuckman A. E.; Batteas J. D.; Drain C. M. Porphyrins as molecular electronic components of functional devices. Coord. Chem. Rev. 2010, 254, 2297–2310. 10.1016/j.ccr.2010.05.014. 20936084 PMC2950646

[ref3] Drobizhev M.; Sigel C.; Rebane A. Photo-tautomer of Br-porphyrin: a new frequency-selective material for ultrafast time–space holographic storage. J. Lumin. 2000, 86, 391–397. 10.1016/S0022-2313(00)00196-4.

[ref4] Teklu Y.; Storm C. B. Nitrogen-hydrogen tautomerism in porphyrins and chlorins. J. Am. Chem. Soc. 1972, 94, 1745–1746. 10.1021/ja00760a056. 5015678

[ref5] Eaton S. S.; Eaton G. R. Kinetic isotope effect on proton tautomerism in tetraarylporphyrins. J. Am. Chem. Soc. 1977, 99, 1601–1604. 10.1021/ja00447a052. 839008

[ref6] Hennig J.; Limbach H. H. Localization and transfer of protons between nitrogen-15 atoms of *meso*-tetraphenylporphine probed by nuclear Overhauser effects and dipole-dipole relaxation times. J. Am. Chem. Soc. 1984, 106, 292–298. 10.1021/ja00314a006.

[ref7] Limbach H. H.; Hennig J.; Kendrick R.; Yannoni C. S. Proton-transfer kinetics in solids: tautomerism in free base porphines by nitrogen-15 CPMAS NMR. J. Am. Chem. Soc. 1984, 106, 4059–4060. 10.1021/ja00326a044.

[ref8] Frydman L.; Olivieri A. C.; Diaz L. E.; Frydman B.; Morin F. G.; Mayne C. L.; Grant D. M.; Adler A. D. High-resolution solid-state carbon-13 NMR spectra of porphine and 5,10,15,20-tetraalkylporphyrins: implications for the nitrogen-hydrogen tautomerization process. J. Am. Chem. Soc. 1988, 110, 336–342. 10.1021/ja00210a002.

[ref9] Ghosh A.; Almloef J. Structure and Stability of *cis*-Porphyrin. J. Phys. Chem. 1995, 99, 1073–1075. 10.1021/j100004a003.

[ref10] Braun J.; Schwesinger R.; Williams P. G.; Morimoto H.; Wemmer D. E.; Limbach H.-H. Kinetic H/D/T isotope and solid state effects on the tautomerism of the conjugate porphyrin monoanion. J. Am. Chem. Soc. 1996, 118, 11101–11110. 10.1021/ja961313q.

[ref11] Vangberg T.; Ghosh A. Monodeprotonated free base porphyrin. J. Phys. Chem. B 1997, 101, 1496–1497. 10.1021/jp963408k.

[ref12] Baker J.; Kozlowski P. M.; Jarzecki A. A.; Pulay P. The inner-hydrogen migration in free base porphyrin. Theor. Chem. Acc. 1997, 97, 59–66. 10.1007/s002140050237.

[ref13] Maity D. K.; Bell R. L.; Truong T. N. Mechanism and quantum mechanical tunneling effects on inner hydrogen atom transfer in free base porphyrin: a direct ab initio dynamics study. J. Am. Chem. Soc. 2000, 122, 897–906. 10.1021/ja9925094.

[ref14] Maity D. K.; Truong T. N. Status of theoretical modeling of tautomerization in free-base porphyrin. J. Porphyrins Phthalocyanines 2001, 05, 289–299. 10.1002/jpp.314.

[ref15] Ghosh A.; Wondimagegn T.; Nilsen H. J. Molecular structures, tautomerism, and carbon nucleophilicity of free-base inverted porphyrins and carbaporphyrins: A density functional theoretical study. J. Phys. Chem. B 1998, 102, 10459–10467. 10.1021/jp983010j.

[ref16] Furuta H.; Ishizuka T.; Osuka A.; Dejima H.; Nakagawa H.; Ishikawa Y. NH Tautomerism of *N*-Confused Porphyrin. J. Am. Chem. Soc. 2001, 123, 6207–6208. 10.1021/ja010237a. 11414867

[ref17] Ishizuka T.; Sakashita R.; Iwanaga O.; Morimoto T.; Mori S.; Ishida M.; Toganoh M.; Takegoshi K.; Osuka A.; Furuta H. NH Tautomerism of *N*-confused porphyrin: Solvent/substituent effects and isomerization mechanism. J. Phys. Chem. A 2020, 124, 5756–5769. 10.1021/acs.jpca.0c04779. 32559101

[ref18] Belair J. P.; Ziegler C. J.; Rajesh C. S.; Modarelli D. A. Photophysical characterization of free-base N-confused tetraphenylporphyrins. J. Phys. Chem. A 2002, 106, 6445–6451. 10.1021/jp025569w.

[ref19] Wehrle B.; Limbach H.-H.; Köcher M.; Ermer O.; Vogel E. ^15^N-CPMAS-NMR Study of the Problem of NH Tautomerism in Crystalline Porphine and Porphycene. Angew. Chem. Int. Ed. Engl. 1987, 26, 934–936. 10.1002/anie.198709341.

[ref20] Ghosh A.; Moulder J.; Bröring M.; Vogel E. X-Ray Photoelectron Spectroscopy of Porphycenes: Charge Asymmetry Across Low-Barrier Hydrogen Bonds. Angew. Chem., Int. Ed. 2001, 40, 431–434. 10.1002/1521-3773(20010119)40:2<431::AID-ANIE431>3.0.CO;2-A. 29712408

[ref21] Pietrzak M.; Shibl M. F.; Bröring M.; Kühn O.; Limbach H.-H. ^1^H/^2^H NMR Studies of Geometric H/D Isotope Effects on the Coupled Hydrogen Bonds in Porphycene Derivatives. J. Am. Chem. Soc. 2007, 129, 296–304. 10.1021/ja065170b. 17212408

[ref22] Kumagai T.; Hanke F.; Gawinkowski S.; Sharp J.; Kotsis K.; Waluk J.; Persson M.; Grill L. Controlling Intramolecular Hydrogen Transfer in a Porphycene Molecule with Single Atoms or Molecules Located Nearby. Nat. Chem. 2014, 6, 41–46. 10.1038/nchem.1804. 24345945

[ref23] Ciąćka P.; Fita P.; Listkowski A.; Kijak M.; Nonell S.; Kuzuhara D.; Yamada H.; Radzewicz C.; Waluk J. Tautomerism in Porphycenes: Analysis of Rate-Affecting Factors. J. Phys. Chem. B 2015, 119, 2292–2301. 10.1021/jp506150r. 25105931

[ref24] Koga D.; Ono T.; Shinjo H.; Hisaeda Y. Hydrogen Bond Engineering Visualized by Picometer-Level Distortion of Planar Porphyrin Isomers. J. Phys. Chem. Lett. 2021, 12, 10429–10436. 10.1021/acs.jpclett.1c03020. 34672583

[ref25] Chang C. K. Synthesis and characterization of alkylated isobacteriochlorins, models of siroheme and sirohydrochlorin. Biochemistry 1980, 19, 1971–1976. 10.1021/bi00550a037. 7378387

[ref26] Dicker A. I. M.; Noort M.; Thijssen H. P. H.; Völker S.; Van Der Waals J. H. Zeeman effect of the S_1⃖_S_0_ transition of the two tautomeric forms of chlorin: a study by photochemical hole burning in an n-hexane host. Chem. Phys. Lett. 1981, 78, 212–218. 10.1016/0009-2614(81)80002-4.

[ref27] Schlabach M.; Rumpel H.; Limbach H. H. Investigation of the Tautomerism of ^15^N-Labeled Hydroporphyrins by Dynamic NMR Spectroscopy. Angew. Chem., Int. Ed. 1989, 28, 76–79. 10.1002/anie.198900761.

[ref28] Huang W. Y.; Wild U. P.; Johnson L. W. Single site spectra of free base isobacteriochlorin in an *n*-octane matrix at 10 K. J. Phys. Chem. 1992, 96, 6189–6195. 10.1021/j100194a020.

[ref29] Solov’ev K. N.; Shkirman S. F. Photoinduced NH-tautomerism and vibronic states of hydroporphyrin molecules. J. Appl. Spectrosc. 1993, 58, 29–35. 10.1007/BF00659156.

[ref30] Almlof J.; Fischer T. H.; Gassman P. G.; Ghosh A.; Haser M. Electron correlation in tetrapyrroles: ab initio calculations on porphyrin and the tautomers of chlorin. J. Phys. Chem. 1993, 97, 10964–10970. 10.1021/j100144a012.

[ref31] Ghosh A. Theoretical Comparative Study of Free Base Porphyrin, Chlorin, Bacteriochlorin, and Isobacteriochlorin: Evaluation of the Potential Roles of *ab Initio* Hartree–Fock and Density Functional Theories in Hydroporphyrin Chemistry. J. Phys. Chem. B 1997, 101, 3290–3297. 10.1021/jp964069y.

[ref32] Helaja J.; Montforts F.-P.; Kilpeläinen I.; Hynninen P. H. NH tautomerism in the dimethyl ester of bonellin, a natural chlorin. J. Org. Chem. 1999, 64, 432–437. 10.1021/jo981260q.

[ref33] Helaja J.; Stapelbroek-Möllmann M.; Kilpeläinen I.; Hynninen P. H. NH tautomerism in the natural chlorin derivatives. J. Org. Chem. 2000, 65, 3700–3707. 10.1021/jo9918955. 10864754

[ref34] Drobizhev M.; Karotki A.; Rebane A. Persistent spectral hole burning by simultaneous two-photon absorption. Chem. Phys. Lett. 2001, 334, 76–82. 10.1016/S0009-2614(00)01459-7.

[ref35] Bruhn T.; Brückner C. Origin of the regioselective reduction of chlorins. J. Org. Chem. 2015, 80, 4861–4868. 10.1021/acs.joc.5b00137. 25719438

[ref36] Meier B. H.; Storm C. B.; Earl W. L. Two-dimensional chemical exchange NMR in the solid: proton dynamics in phthalocyanine. J. Am. Chem. Soc. 1986, 108, 6072–6074. 10.1021/ja00279a084. 22175392

[ref37] Wehrle B.; Limbach H. H. NMR study of environment modulated proton tautomerism in crystalline and amorphous phthalocyanine. Chem. Phys. 1989, 136, 223–247. 10.1016/0301-0104(89)80049-7.

[ref38] Rebane A.; Drobizhev M.; Sigel C. Single femtosecond exposure recording of an image hologram by spectral hole burning in an unstable tautomer of a phthalocyanine derivative. Opt. Lett. 2000, 25, 1633–1635. 10.1364/OL.25.001633. 18066298

[ref39] Liljeroth P.; Repp J.; Meyer G. Current-induced hydrogen tautomerization and conductance switching of naphthalocyanine molecules. Science 2007, 317, 1203–1206. 10.1126/science.1144366. 17761878

[ref40] Toganoh M.; Furuta H. Theoretical Study on Conformation and Electronic State of Hückel-Aromatic Multiply *N*-Confused [26]Hexaphyrins. J. Org. Chem. 2010, 75, 8213–8223. 10.1021/jo101856h. 21058649

[ref41] Mack J. Expanded, contracted, and isomeric porphyrins: Theoretical aspects. Chem. Rev. 2017, 117, 3444–3478. 10.1021/acs.chemrev.6b00568. 28222605

[ref42] Ghosh A.; Jynge K. Molecular structures and energetics of corrole isomers: a comprehensive local density functional theoretical study. Chem. – Eur. J. 1997, 3, 823–833. 10.1002/chem.19970030523.

[ref43] Ivanova Y. B.; Savva V. A.; Mamardashvili N. Z.; Starukhin A. S.; Ngo T. H.; Dehaen W.; Maes W.; Kruk M. M. Corrole NH tautomers: spectral features and individual protonation. J. Phys. Chem. A 2012, 116, 10683–10694. 10.1021/jp305325e. 22985133

[ref44] Kruk M.; Ngo T. H.; Verstappen P.; Starukhin A.; Hofkens J.; Dehaen W.; Maes W. Unraveling the fluorescence features of individual corrole NH tautomers. J. Phys. Chem. A 2012, 116, 10695–10703. 10.1021/jp305326x. 22985194

[ref45] Ding T.; Harvey J. D.; Ziegler C. J. N-H tautomerization in triaryl corroles. J. Porphyrins Phthalocyanines 2005, 09, 22–27. 10.1142/S1088424605000058.

[ref46] Beenken W.; Presselt M.; Ngo T. H.; Dehaen W.; Maes W.; Kruk M. Molecular structures and absorption spectra assignment of corrole NH tautomers. J. Phys. Chem. A 2014, 118, 862–871. 10.1021/jp411033h. 24432802

[ref47] Gladkov L. L.; Klenitsky D. V.; Vershilovskaya I. V.; Maes W.; Kruk M. M. Inversion of Aromaticity of NH-Tautomers of Free-Base Corroles in the Lowest Triplet T_1_-State. J. Appl. Spectrosc. 2022, 89, 426–432. 10.1007/s10812-022-01374-w.

[ref48] Johnson A. W.; Kay I. T. 306. Corroles. Part I. Synthesis. J. Chem. Soc. 1965, 79, 1620–1629. 10.1039/jr9650001620.

[ref49] Paolesse R.; Mini S.; Sagone F.; Boschi T.; Jaquinod L.; Nurco D. J.; Smith K. M. 5,10,15-Triphenylcorrole: a product from a modified Rothemund reaction. Chem. Commun. 1999, 1307–1308. 10.1039/a903247i.

[ref50] Paolesse R.; Marini A.; Nardis S.; Froiio A.; Mandoj F.; Nurco D. J.; Prodi L.; Montalti M.; Smith K. M. Novel routes to substituted 5, 10, 15-triarylcorroles. J. Porphyrins Phthalocyanines 2003, 07, 25–36. 10.1142/S1088424603000057.

[ref51] Ou Z.; Sun H.; Zhu W.; Da Z.; Kadish K. M. Solvent and acidity effects on the UV-visible spectra and protonation-deprotonation of free-base octaethylcorrole. J. Porphyrins Phthalocyanines 2008, 12, 1–10. 10.1142/S1088424608000029.

[ref52] Ajeeb Y. H.; Minchenya A. A.; Klimovich P. G.; Maes W.; Kruk M. M. Thermochromism of corrole solutions in ethanol. J. Appl. Spectrosc. 2019, 86, 788–794. 10.1007/s10812-019-00894-2.

[ref53] Mahammed A.; Weaver J. J.; Gray H. B.; Abdelas M.; Gross Z. How acidic are corroles and why?. Tetrahedron Lett. 2003, 44, 2077–2079. 10.1016/S0040-4039(03)00174-6.

[ref54] Yadav P.; Sankar M. Spectroscopic and theoretical studies of anionic corroles derived from phosphoryl and carbomethoxyphenyl substituted corroles. Chem. Phys. Lett. 2017, 677, 107–113. 10.1016/j.cplett.2017.04.011.

[ref55] Shen J.; Shao J.; Ou Z.; E W.; Koszarna B.; Gryko D. T.; Kadish K. M. Electrochemistry and spectroelectrochemistry of *meso*-substituted free-base corroles in nonaqueous media: reactions of (Cor)H_3_, [(Cor)H_4_]^+^, and [(Cor)H_2_]^−^. Inorg. Chem. 2006, 45, 2251–2265. 10.1021/ic051729h. 16499391

[ref56] Shen J.; Ou Z.; Shao J.; Gałęzowski M.; Gryko D. T.; Kadish K. M. Free-base corroles: determination of deprotonation constants in non-aqueous media. J. Porphyrins Phthalocyanines 2007, 11, 269–276. 10.1142/S1088424607000321.

[ref57] Song Y.; Fang Y.; Ou Z.; Capar J.; Wang C.; Conradie J.; Thomas K. E.; Wamser C. C.; Ghosh A.; Kadish K. M. Influence of *β*-octabromination on free-base triarylcorroles: Electrochemistry and protonation-deprotonation reactions in nonaqueous media. J. Porphyrins Phthalocyanines 2017, 21, 633–645. 10.1142/S1088424617500602.

[ref58] Clark J. A.; Orłowski R.; Derr J. B.; Espinoza E. M.; Gryko D. T.; Vullev V. I. How does tautomerization affect the excited-state dynamics of an amino acid-derivatized corrole?. Photosynth. Res. 2021, 148, 67–76. 10.1007/s11120-021-00824-4. 33710530 PMC8154756

[ref59] Capar J.; Conradie J.; Beavers C. M.; Ghosh A. Molecular structures of free-base corroles: nonplanarity, chirality, and enantiomerization. J. Phys. Chem. A 2015, 119, 3452–3457. 10.1021/jp511188c. 25819028

[ref60] Orłowski R.; Tasior M.; Staszewska-Krajewska O.; Dobrzycki Ł.; Schilf W.; Ventura B.; Cyrański M. K.; Gryko D. T. Hydrogen Bonds Involving Cavity NH Protons Drives Supramolecular Oligomerization of Amido-Corroles. Chem. – Eur. J. 2017, 23, 10195–10204. 10.1002/chem.201701674. 28514507

[ref61] Orłowski R.; Cichowicz G.; Staszewska-Krajewska O.; Schilf W.; Cyrański M. K.; Gryko D. T. Covalently Linked Bis(Amido-Corroles): Inter- and Intramolecular Hydrogen-Bond-Driven Supramolecular Assembly. Chem. – Eur. J. 2019, 25, 9658–9664. 10.1002/chem.201901254. 30990230

[ref62] Koszarna B.; Gryko D. T. Efficient synthesis of *meso*-substituted corroles in a H_2_O–MeOH mixture. J. Org. Chem. 2006, 71, 3707–3717. 10.1021/jo060007k. 16674040

[ref63] Gryko D. T.; Koszarna B. Refined methods for the synthesis of *meso*-substituted A_3_-and *trans*-A_2_B-corroles. Org. Biomol. Chem. 2003, 1, 350–357. 10.1039/b208950e. 12929430

[ref64] Etter M. C. Encoding and decoding hydrogen-bond patterns of organic compounds. Acc. Chem. Res. 1990, 23, 120–126. 10.1021/ar00172a005.

[ref65] Thomas K. E.; McCormick L. J.; Vazquez-Lima H.; Ghosh A. Stabilization and Structure of the *cis* Tautomer of a Free-Base Porphyrin. Am. Ethnol. 2017, 56, 10088–10092. 10.1002/anie.201701965. 28370984

[ref66] Thomassen I. K.; McCormick L. J.; Ghosh A. Molecular structure of a free-base *β*-Octaiodo-*meso*-tetraarylporphyrin. A rational route to *cis* porphyrin tautomers?. Cryst. Growth Des. 2018, 18, 4257–4259. 10.1021/acs.cgd.8b00629.

[ref67] Thomas K. E.; Slebodnick C.; Ghosh A. Facile Supramolecular Engineering of Porphyrin cis Tautomers: The Case of *β*-Octabromo-*meso*-tetraarylporphyrins. ACS Omega 2020, 5, 8893–8901. 10.1021/acsomega.0c00517. 32337452 PMC7178774

[ref68] Thomas K. E.; Conradie J.; Beavers C. M.; Ghosh A. Free-base porphyrins with localized NH protons. Can substituents alone stabilize the elusive *cis* tautomer?. Org. Biomol. Chem. 2020, 18, 2861–2865. 10.1039/D0OB00452A. 32215434

[ref69] Kielmann M.; Senge M. O. Molecular engineering of free-base porphyrins as ligands—the N– H···X binding motif in tetrapyrroles. Angew. Chem., Int. Ed. 2019, 58, 418–441. 10.1002/anie.201806281. PMC6391963 30067890

[ref70] Gerlt J. A.; Gassman P. G. Understanding the rates of certain enzyme-catalyzed reactions: Proton abstraction from carbon acids, acyl transfer reactions, and displacement reactions of phosphodiesters. Biochemistry 1993, 32, 11943–11952. 10.1021/bi00096a001. 8218268

[ref71] Cleland W. W.; Kreevoy M. M. Low-barrier hydrogen bonds and enzymic catalysis. Science 1994, 264, 1887–1890. 10.1126/science.8009219. 8009219

[ref72] Cleland W. W.; Frey P. A.; Gerlt J. A. The low barrier hydrogen bond in enzymatic catalysis. J. Biol. Chem. 1998, 273, 25529–25532. 10.1074/jbc.273.40.25529. 9748211

[ref73] Klinman J. P. Low Barrier Hydrogen Bonds: Getting Close, but Not Sharing. ACS Cent. Sci. 2015, 1, 115–116. 10.1021/acscentsci.5b00215. 27162960 PMC4827480

[ref74] Schutz C. N.; Warshel A. The low barrier hydrogen bond (LBHB) proposal revisited: The case of the Asp··· His pair in serine proteases. Proteins: Struct., Funct., Bioinf. 2004, 55, 711–723. 10.1002/prot.20096. 15103633

[ref75] Perrin C. L. Are short, low-barrier hydrogen bonds unusually strong?. Acc. Chem. Res. 2010, 43, 1550–1557. 10.1021/ar100097j. 20939528

[ref76] Oltrogge L. M.; Boxer S. G. Short hydrogen bonds and proton delocalization in green fluorescent protein (GFP). ACS Cent. Sci. 2015, 1, 148–156. 10.1021/acscentsci.5b00160. 27162964 PMC4827562

[ref77] Dai S.; Funk L. M.; von Pappenheim F. R.; Sautner V.; Paulikat M.; Schröder B.; Uranga J.; Mata R. A.; Tittmann K. Low-barrier hydrogen bonds in enzyme cooperativity. Nature 2019, 573, 609–613. 10.1038/s41586-019-1581-9. 31534226

[ref78] Schiøtt B.; Iversen B. B.; Hellerup Madsen G. K.; Bruice T. C. Characterization of the short strong hydrogen bond in benzoylacetone by ab initio calculations and accurate diffraction experiments. Implications for the electronic nature of low-barrier hydrogen bonds in enzymatic reactions. J. Am. Chem. Soc. 1998, 120, 12117–12124. 10.1021/ja982317t.

[ref79] Garcia-Viloca M.; González-Lafont A.; Lluch J. M. Theoretical study of the low-barrier hydrogen bond in the hydrogen maleate anion in the gas phase. Comparison with normal hydrogen bonds. J. Am. Chem. Soc. 1997, 119, 1081–1086. 10.1021/ja962662n.

[ref80] Kong X.; Brinkmann A.; Terskikh V.; Wasylishen R. E.; Bernard G. M.; Duan Z.; Wu Q.; Wu G. Proton probability distribution in the O··· H··· O low-barrier hydrogen bond: A combined solid-state NMR and quantum chemical computational study of dibenzoylmethane and curcumin. J. Phys. Chem. B 2016, 120, 11692–11704. 10.1021/acs.jpcb.6b08091. 27782387

[ref81] Rurack K.; Spieles M. Fluorescence quantum yields of a series of red and near-infrared dyes emitting at 600-1000 nm. Anal. Chem. 2011, 83, 1232–1242. 10.1021/ac101329h. 21250654

[ref82] Sheldrick G. M. SHELXT - Integrated Space-Group and Crystal-Structure Determination. Acta Crystallogr., Sect. A: Found. Adv. 2015, 71, 3–8. 10.1107/S2053273314026370. 25537383 PMC4283466

[ref83] Sheldrick G. M. Crystal Structure Refinement with SHELXL. Acta Cryst.allogr., Sect. C: Struct. Chem. 2015, 71, 3–8. 10.1107/S2053229614024218. PMC4294323 25567568

[ref84] Spek A. L. PLATON SQUEEZE: a tool for the calculation of the disordered solvent contribution to the calculated structure factors. Acta Crystallogr., Sect. C: Struct. Chem. 2015, C71, 9–18. 10.1107/S2053229614024929. 25567569

