# Supplementary material for: Free-Base Corrole Anion
Source: J Org Chem. 2023 Aug 30;88(18):13022–9. doi: 10.1021/acs.joc.3c01125 (PMC10763984; doi:10.1021/acs.joc.3c01125)
Supplement: Supplementary file 1 — jo3c01125_si_001.pdf [file jo3c01125_si_001.pdf]

Supporting Information

Free-Base Corrole Anion

Arup Tarai,[†] Jyotiprakash Mallick,[†] Pranjali Singh,[†] Jeanet Conradie,*[,‡,ʃ] Sanjib Kar*[,†], Abhik Ghosh*[,ʃ]

[†]*School of Chemical Sciences, National Institute of Science Education and Research (NISER), Bhubaneswar – 752050, India, and Homi Bhabha National Institute, Training School Complex, Anushakti Nagar, Mumbai, 400 094, India. E-mail: sanjib@niser.ac.in (SK)*

[‡]*Department of Chemistry, University of the Free State, P.O. Box 339, Bloemfontein 9300, Republic of South Africa. E-mail: conradj@ufs.ac.za (JC)*

[ʃ]*Department of Chemistry, UiT – The Arctic University of Norway, N-9037 Tromsø, Norway;*
Email: abhik.ghosh@uit.no (AG)

Contents

Figures S3

Tables S15

Optimized Cartesian coordinates (Å): S17

Isomer BD S17

Isomer AC S18

Isomer AD S20

Isomer BC S21

Isomer AB S23

Isomer CD S24

Transition state A-BC‡ S26

Transition state AB-C‡ S28

Transition state AC-D‡ S29

Transition state CD-A‡ S31

Figure S1.	FT-IR spectrum of H_3Cor [Cor = 5,10,15-tris(4-cyanophenyl)corrole].
Figure S2.	FT IR spectrum of TBA[H_2Cor].
Figure S3.	ESI mass spectrum of H_3Cor.
Figure S4.	ESI mass spectrum of TBA[H_2Cor].
Figure S5.	^{1}H NMR spectrum ($CDCl_3$, 400 MHz) of H_3Cor.
Figure S6.	^{1}H NMR spectrum ($CDCl_3$, 400 MHz) of TBA[H_2Cor].
Figure S7.	^{13}C{^{1}H} NMR spectrum ($CDCl_3$, 400 MHz) of TBA[H_2Cor].
Figure S8.	Hydrogen bonding interactions between [H_2Cor] and TBA (Some hydrogen atoms have been omitted for clarity).
Figure S9.	The TBA cation encapsulated by supramolecular assemblies of [H_2Cor].
Figure S10.	Chain-like supramolecular assemblies of [H_2Cor] formed by hydrogen bonding interactions.
Figure S11.	Time-resolved fluorescence spectrum of H_3Cor in dichloromethane.
Figure S12.	Time-resolved fluorescence spectrum of TBA[H_2Cor] in dichloromethane.
Table S1	Crystallographic parameters for TBA[H_2Cor].
Table S2	Some important hydrogen bond parameters of TBA[H_2Cor].

Figures

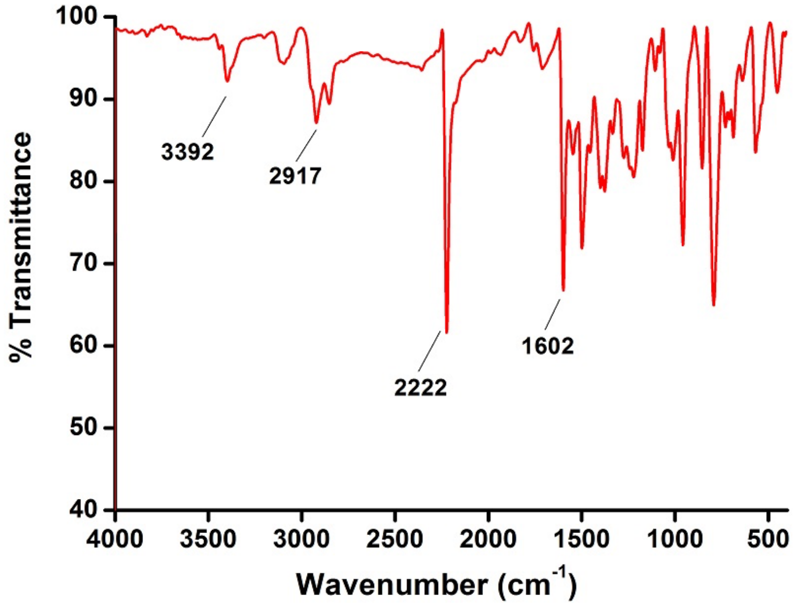

Figure S1. FT-IR spectrum of H_3Cor [Cor = 5,10,15-tris(4-cyanophenyl)corrole].

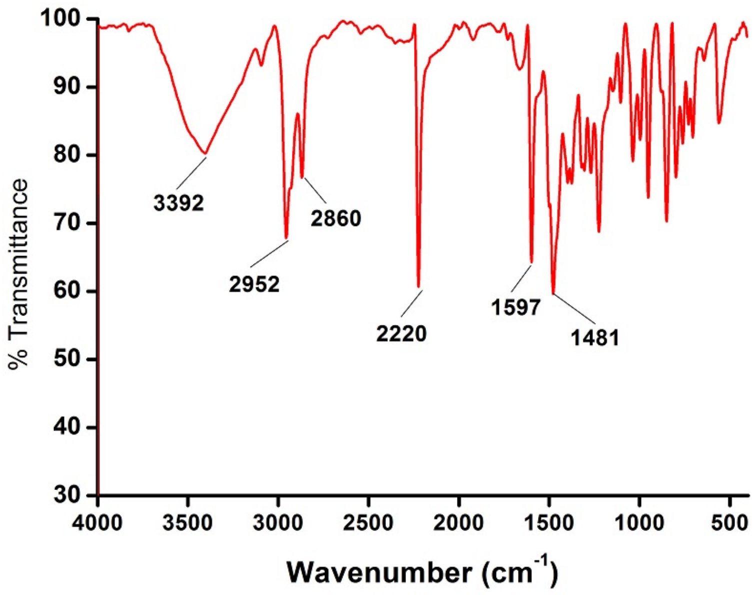

Figure S2. FT IR spectrum of TBA[H_2Cor].

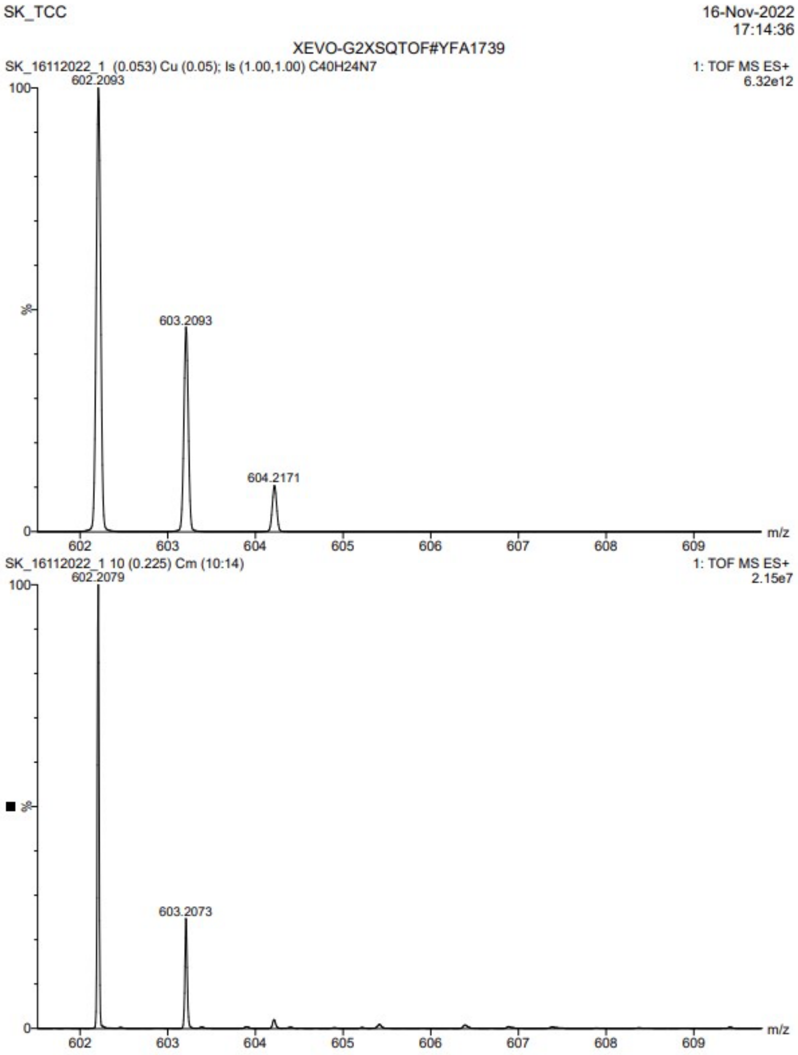

Figure S3. ESI mass spectrum of H_3Cor.

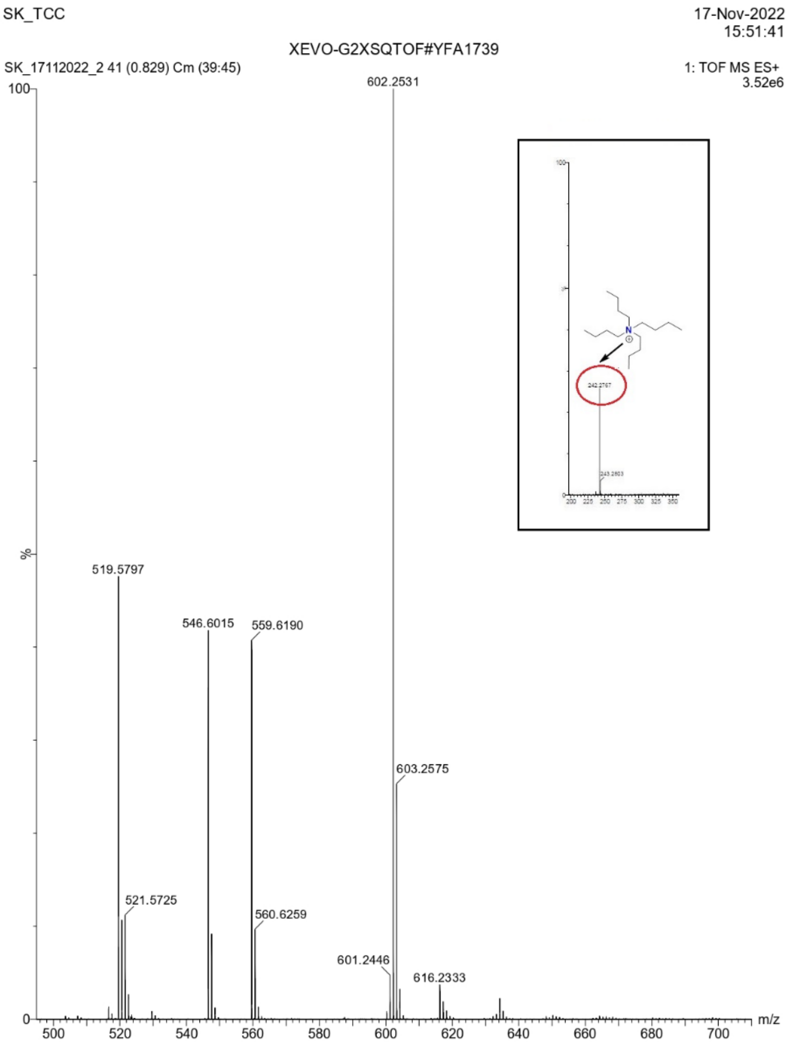

Figure S4. ESI mass spectrum of TBA[H_2Cor].

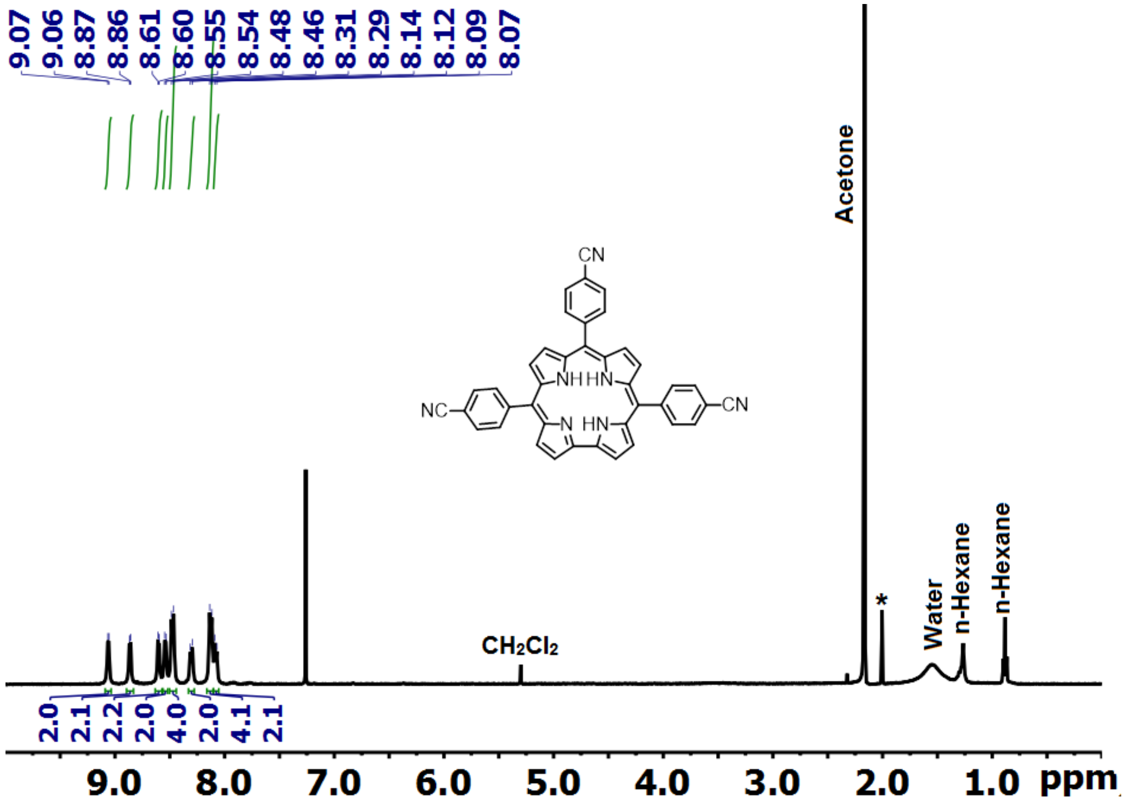

Figure S5. ^{1}H NMR spectrum ($CDCl_3$, 400 MHz) of H_3Cor. Impurity peaks were assigned based on the previous reports (see ref: Fulmer, G. R.; Miller, A. J.; Sherden, N. H.; Gottlieb, H. E.; Nudelman, A.; Stoltz, B. M.; Bercaw, J. E.; Goldberg, K. I. NMR chemical shifts of trace impurities: common laboratory solvents, organics, and gases in deuterated solvents relevant to the organometallic chemist. *Organometallics*, **2010**, *29*, 2176-2179).

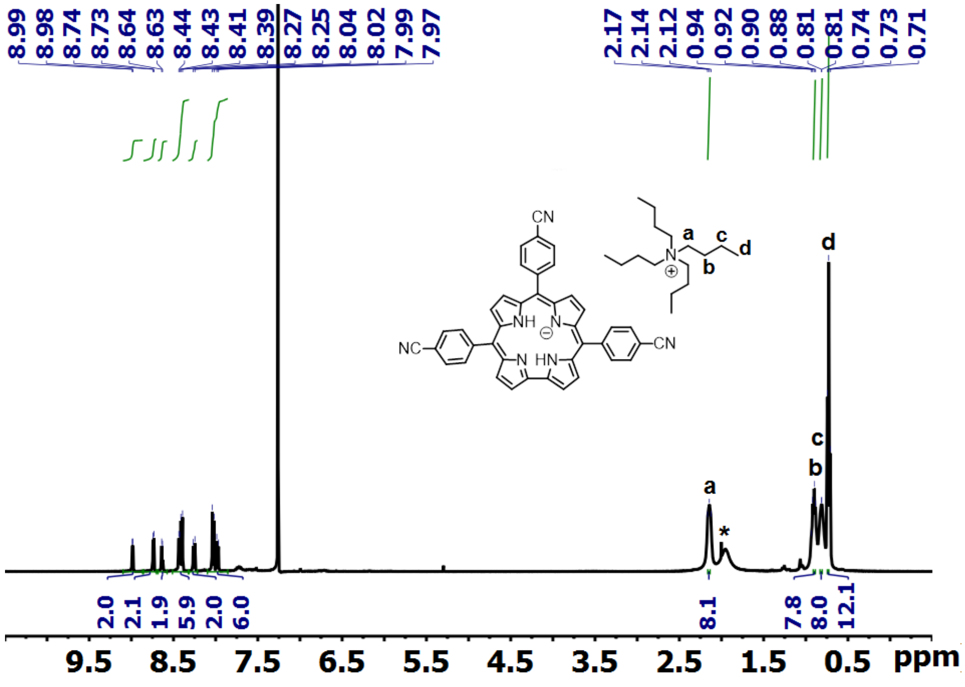

Figure S6. ^{1}H NMR spectrum ($CDCl_3$, 400 MHz) of **TBA[H_2Cor]**. The asterisk mark (*) corresponds to a peak position due to the cluster states of water molecules in $CDCl_3$ (see ref: Nakahara, M.; Wakai, C. Monomeric and cluster states of water molecules in organic solvent. *Chem. Lett.* **1992**, *21*, 809-812).

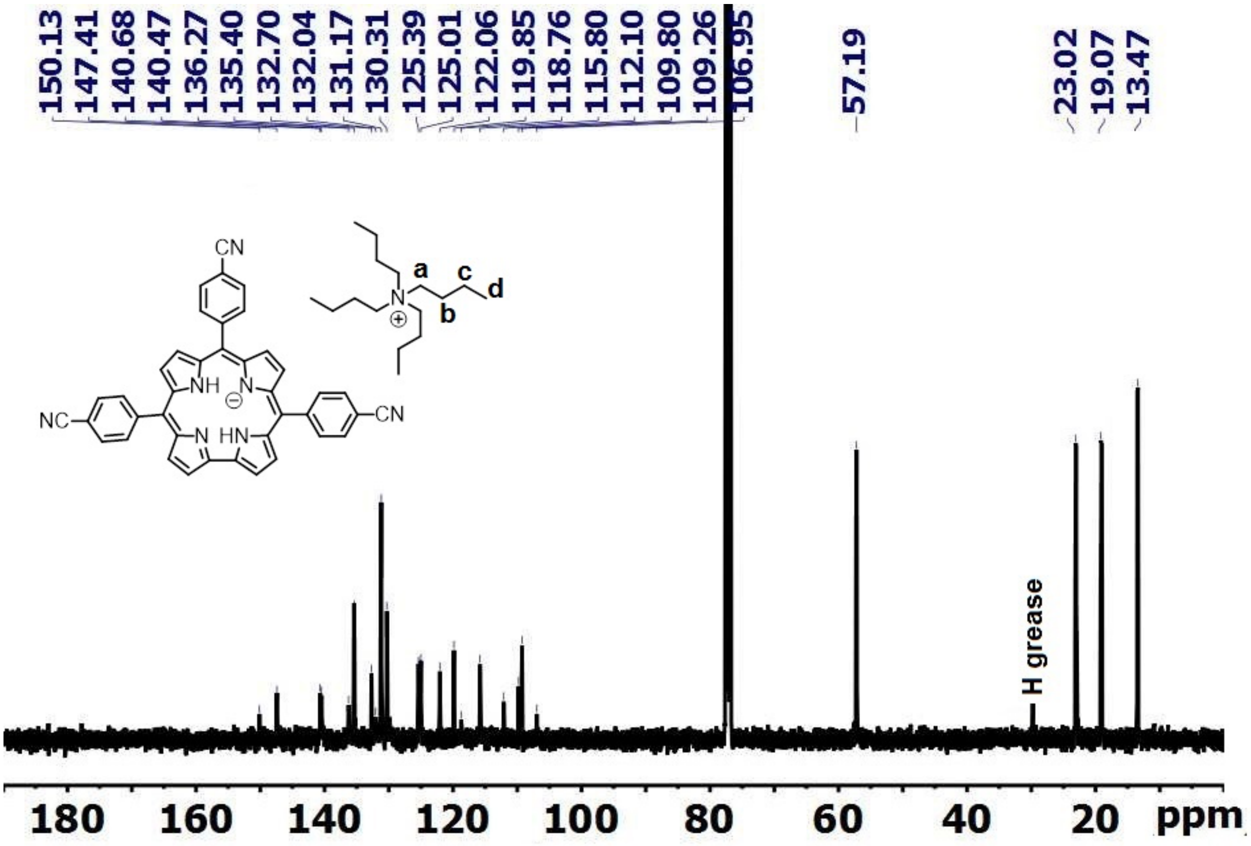

Figure S7 ^{13}C {1H} NMR spectrum (101 MHz, $CDCl_3$) of **TBA[H_2Cor]**. The extra peak is assigned based on the previous reports (see ref: Fulmer, G. R.; Miller, A. J.; Sherden, N. H.; Gottlieb, H. E.; Nudelman, A.; Stoltz, B. M.; Bercaw, J. E.; Goldberg, K. I. NMR chemical shifts of trace impurities: common laboratory solvents, organics, and gases in deuterated solvents relevant to the organometallic chemist. *Organometallics*, **2010**, *29*, 2176-2179).

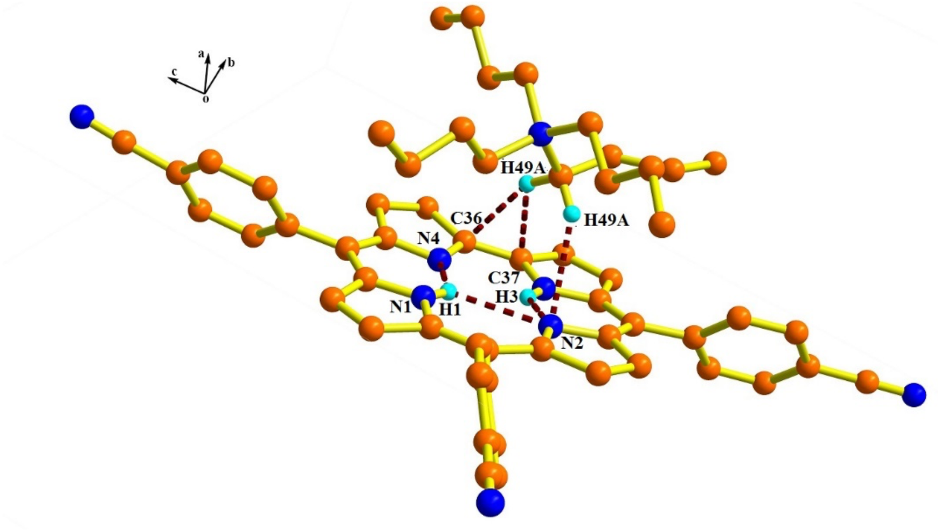

Figure S8. Hydrogen bonding interactions between H_2Cor^- and TBA^+ (Some hydrogen atoms have been omitted for clarity).

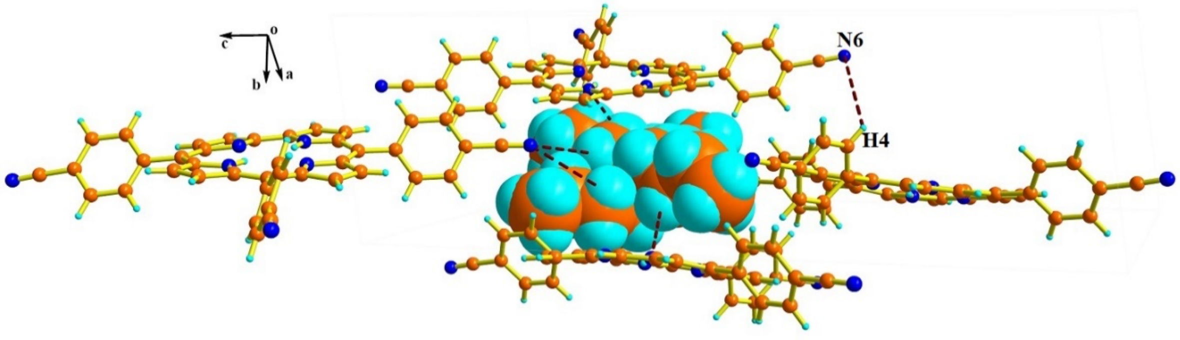

Figure S9. The TBA cation encapsulated by supramolecular assemblies of H_2Cor^-.

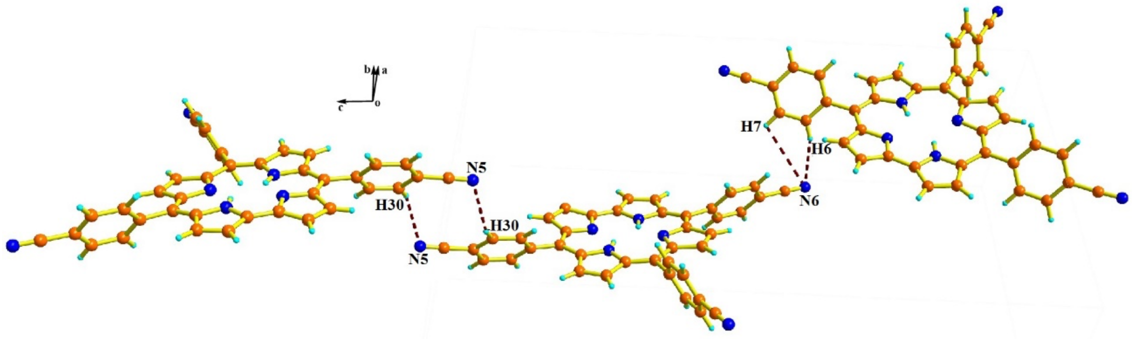

Figure S10. Chain-like supramolecular assemblies of [H_2Cor] formed by hydrogen bonding interactions.

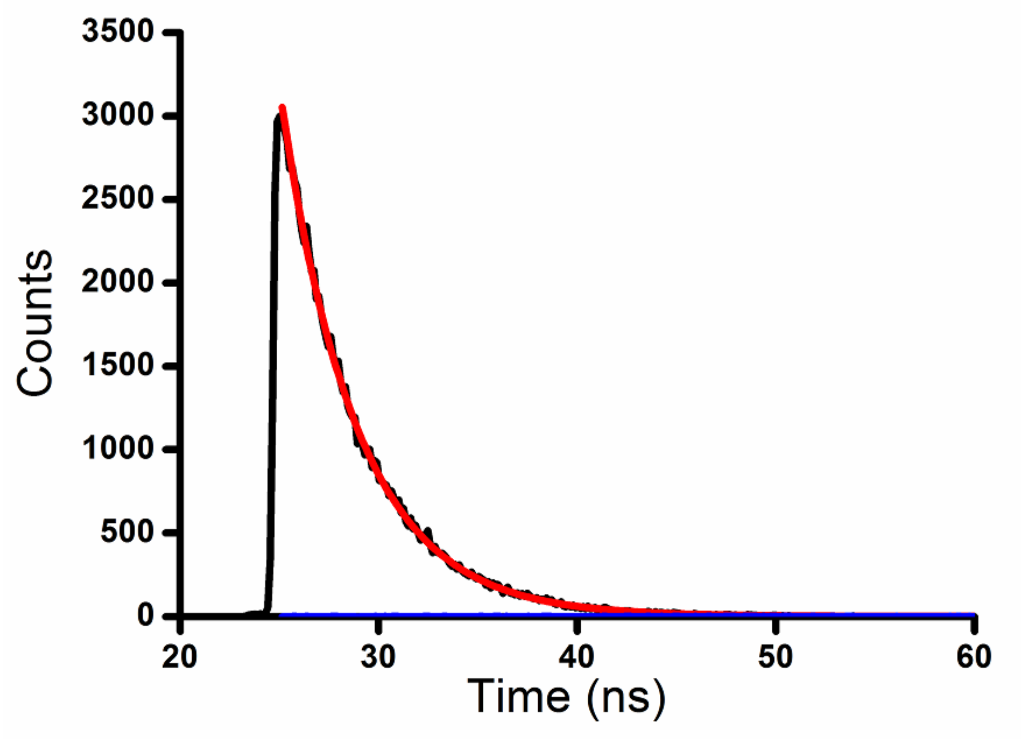

Figure S11. Time-resolved fluorescence spectrum of H_3Cor in dichloromethane.

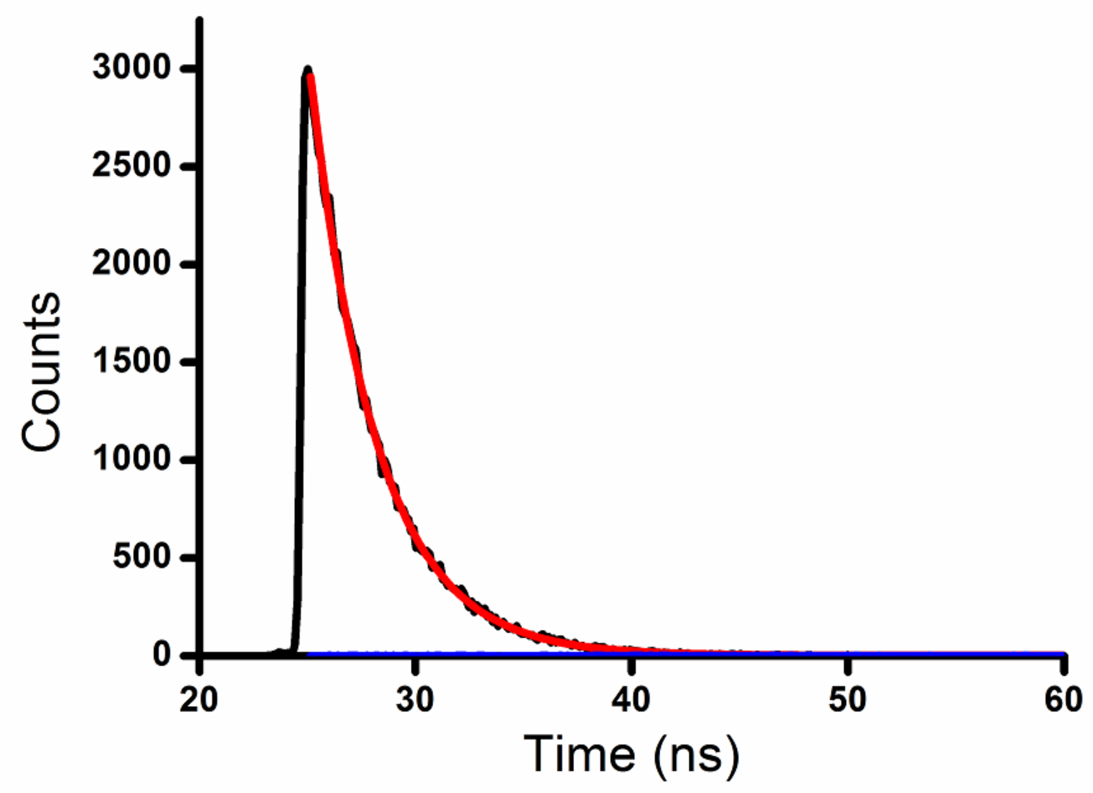

Figure S12. Time-resolved fluorescence spectrum of TBA[H_2Cor] in dichloromethane.

Tables

Table S1 Crystallographic parameters for TBA[H_2Cor].

Compound	TBA[H_2Cor]
Chemical formula	$C_{56}H_{58}N_8$
Formula mass	843.08
Crystal system	Monoclinic
Crystal size (mm)	0.31 x 0.22 x 0.12
Space group	*P* 1 2_1/n 1
Radiation	Cu Kα
a (Å)	8.5424(3)
b (Å)	20.0707(7)
c (Å)	27.6314(11)
α (º)	90
β (º)	97.367(4)
γ (º)	90
V (Å^3)	4698.3(3)
Z	4
T (K)	100
D_{calcd} (g cm^{-3})	1.189
Measured reflections	14853
e data (R_{int})	9935 (0.048)
Parameters	600
Restraints	0
μ (mm^{-1})	0.071
2Θ range (º)	3.64-30.51
R_1 (I > 2σ (I))	0.0577
wR_2 (All data)	0.1539
S (GooF) all data	1.021
$\Delta\rho_{max}$, $\Delta\rho_{min}$ (e Å^{-3})	1.20, -0.34

Table S2 Some important hydrogen bond parameters of TBA[H_2Cor].

Compound	D-H···A	^{d}D-H (Å)	^{d}H···A (Å)	^{d}D···A (Å)	∠D-H···A (°)
TBA[H_2Cor]	N(1)-H(1)…N(2)	0.85	2.483	2.969	116.88
	N(1)-H(1)…N(4)	0.85	1.971	2.616	131.51
	N(3)-H(3)…N(2)	0.87	1.894	2.606	137.59
	C(30)-H(30)…N(5)	0.95	2.706	3.418	132.19

Optimized Cartesian coordinates (Å):

All compounds were optimized the OLYP functional augmented with D3 dispersion corrections, using the ZORA Hamiltonian, ZORA/TZ2P all electron basis set.

Isomer BD

C	0.013041000	2.547028000	1.764008000
C	0.046238000	-0.012764000	1.936196000
C	0.123931000	-2.551048000	2.098005000
C	1.513584000	0.024769000	2.142185000
C	2.096790000	-0.522197000	3.301068000
C	2.373953000	0.615546000	1.197882000
C	3.467858000	-0.486594000	3.511475000
C	3.747814000	0.661167000	1.392709000
C	4.315160000	0.108363000	2.556910000
C	5.724076000	0.151545000	2.770246000
C	-0.578327000	1.241039000	1.716796000
C	-0.588048000	-1.286478000	1.990499000
C	-0.815616000	-3.541725000	2.051507000
C	-0.980620000	3.486029000	1.574918000
C	-2.102914000	-2.893153000	1.904406000
C	-2.220405000	2.796720000	1.412434000
C	-2.650065000	-5.761460000	0.858008000
C	-2.774684000	-7.137462000	0.740634000
C	-3.003512000	5.493463000	0.180922000
C	-3.225546000	6.848652000	-0.011557000
C	-3.364984000	-3.556347000	1.840426000
C	-3.449060000	-5.020884000	1.754173000
C	-3.541853000	3.305178000	1.301885000
C	-3.714159000	-7.837033000	1.523660000
C	-3.746015000	4.748677000	1.120781000
C	-3.856955000	-9.248451000	1.389884000
C	-4.207171000	7.522350000	0.741824000
C	-4.380833000	-5.737911000	2.535105000
C	-4.439974000	8.914801000	0.545618000
C	-4.514435000	-7.114176000	2.429961000
C	-4.581662000	-2.824723000	1.799283000
C	-4.671970000	2.442788000	1.360110000
C	-4.721987000	5.439745000	1.870459000
C	-4.951938000	6.795125000	1.691152000
C	-5.723228000	0.526098000	1.478682000
C	-5.739130000	-0.888738000	1.611739000
C	-5.937162000	-3.150161000	1.479351000
C	-6.064833000	2.710183000	1.050220000
C	-6.647163000	-1.954593000	1.368634000
C	-6.722699000	1.499222000	1.127463000

H	1.058122000	2.743045000	1.948190000
H	1.194172000	-2.673594000	2.176903000
H	1.448051000	-0.974002000	4.044354000
H	1.943579000	1.036718000	0.294901000
H	3.895738000	-0.905171000	4.418261000
H	4.394718000	1.112395000	0.645600000
H	-0.645496000	-4.606267000	2.126927000
H	-0.868332000	4.559955000	1.586199000
H	-1.944735000	-5.229551000	0.228657000
H	-2.161591000	-7.684377000	0.029509000
H	-2.260998000	4.979536000	-0.420591000
H	-2.654626000	7.398000000	-0.755073000
H	-2.671942000	0.766959000	1.525748000
H	-3.639942000	-0.991109000	1.987560000
H	-4.997022000	-5.189883000	3.240573000
H	-5.232617000	-7.644783000	3.049327000
H	-5.294416000	4.888634000	2.609186000
H	-5.702772000	7.307364000	2.286821000
H	-6.317991000	-4.147625000	1.315747000
H	-6.489687000	3.667095000	0.778938000
H	-7.692765000	-1.843108000	1.114047000
H	-7.774494000	1.309293000	0.948589000
N	6.875495000	0.187614000	2.949188000
N	-1.915305000	1.451531000	1.487361000
N	-1.929159000	-1.524923000	1.881077000
N	-3.971634000	-10.402921000	1.271644000
N	-4.513963000	1.118185000	1.620330000
N	-4.544934000	-1.469023000	1.913180000
N	-4.624278000	10.054734000	0.382819000

Isomer AC

C	0.038976000	-0.006141000	1.956046000
C	0.178840000	-2.570119000	1.964498000
C	1.507332000	0.073906000	2.146608000
C	2.133526000	-0.533751000	3.251373000
C	2.325872000	0.771524000	1.238248000
C	3.504213000	-0.444592000	3.451390000
C	3.698014000	0.871257000	1.423015000
C	4.307686000	0.264777000	2.537889000
C	5.714566000	0.375465000	2.743618000
C	-0.060574000	2.538912000	1.979963000
C	-0.512261000	-1.312980000	1.930777000
C	-0.667149000	1.224206000	1.828314000
C	-0.754902000	-3.586491000	1.911683000

C	-1.053733000	3.458808000	1.795845000
C	-2.051486000	-2.994445000	1.825668000
C	-2.273211000	2.715952000	1.547285000
C	-2.727259000	-5.820534000	0.792622000
C	-2.883494000	5.426416000	0.246437000
C	-2.896033000	-7.193768000	0.690491000
C	-3.051722000	6.777857000	-0.012825000
C	-3.340724000	-3.589047000	1.787197000
C	-3.467547000	-5.052277000	1.714486000
C	-3.571084000	3.292224000	1.380873000
C	-3.723771000	4.732312000	1.144817000
C	-3.818657000	-7.860761000	1.520483000
C	-4.007643000	-9.269130000	1.407921000
C	-4.081407000	7.502272000	0.620263000
C	-4.270095000	8.887166000	0.342365000
C	-4.380195000	-5.736865000	2.544325000
C	-4.521132000	-2.794781000	1.773957000
C	-4.556181000	-7.109628000	2.456252000
C	-4.739440000	2.483030000	1.415354000
C	-4.744578000	5.476295000	1.777178000
C	-4.924747000	6.828038000	1.525999000
C	-5.681911000	-0.942610000	1.631834000
C	-5.775575000	0.472236000	1.533310000
C	-5.896058000	-3.180929000	1.508719000
C	-6.121573000	2.678861000	1.103950000
C	-6.622651000	-2.011050000	1.422671000
C	-6.756563000	1.438783000	1.183658000
H	0.972609000	2.739022000	2.223745000
H	1.251179000	-2.681181000	2.011804000
H	1.518421000	-1.069845000	3.966976000
H	1.861589000	1.239550000	0.376476000
H	3.963931000	-0.906958000	4.320521000
H	4.311200000	1.411156000	0.706861000
H	-0.559341000	-4.648036000	1.938676000
H	-0.971650000	4.534071000	1.870213000
H	-2.034882000	-5.313685000	0.128531000
H	-2.105392000	4.873185000	-0.268333000
H	-2.331057000	-7.762623000	-0.042839000
H	-2.401917000	7.284247000	-0.721378000
H	-2.651673000	-1.005443000	1.823661000
H	-3.674097000	0.749625000	1.783470000
H	-4.950014000	-5.165306000	3.269554000
H	-5.260487000	-7.615643000	3.111021000
H	-5.390502000	4.972936000	2.488935000
H	-5.712132000	7.379489000	2.032900000
H	-6.263013000	-4.188151000	1.366227000
H	-6.575576000	3.620220000	0.831191000

H	-7.680874000	-1.907957000	1.213835000
H	-7.802451000	1.233251000	0.997403000
N	6.863786000	0.471349000	2.914628000
N	-1.842839000	-1.630138000	1.838386000
N	-2.005953000	1.363989000	1.588009000
N	-4.163123000	-10.420667000	1.310350000
N	-4.423380000	10.020444000	0.113058000
N	-4.443514000	-1.439394000	1.860862000
N	-4.610954000	1.157097000	1.693371000

Isomer AD

C	0.114078000	-2.553574000	2.028640000
C	1.430481000	0.046450000	2.127647000
C	2.035730000	-0.501190000	3.274859000
C	2.274391000	0.655584000	1.179897000
C	3.409641000	-0.446770000	3.472062000
C	3.650420000	0.718167000	1.359578000
C	4.239961000	0.166714000	2.513561000
C	5.653126000	0.228504000	2.710127000
C	-0.042534000	-0.010396000	1.928224000
C	-0.051504000	2.538311000	1.856802000
C	-0.633917000	-1.305397000	1.947730000
C	-0.699829000	1.237782000	1.739263000
C	-0.794725000	-3.573910000	1.978498000
C	-1.012176000	3.488456000	1.649385000
C	-2.100552000	-2.960153000	1.849801000
C	-2.252689000	2.778444000	1.413738000
C	-2.632074000	-5.846474000	0.872598000
C	-2.733981000	-7.227943000	0.801061000
C	-2.901137000	5.550702000	0.233020000
C	-3.075491000	6.916217000	0.061442000
C	-3.364920000	-3.618849000	1.789907000
C	-3.457442000	-5.084009000	1.727701000
C	-3.552192000	3.351178000	1.268643000
C	-3.680804000	-7.916223000	1.585787000
C	-3.722383000	4.799707000	1.103473000
C	-3.787950000	-9.336898000	1.513848000
C	-4.092439000	7.600121000	0.757897000
C	-4.286550000	9.001982000	0.577674000
C	-4.405229000	-5.789206000	2.501572000
C	-4.518495000	-7.170526000	2.439059000
C	-4.567508000	-2.857672000	1.746624000
C	-4.706568000	2.517725000	1.307079000
C	-4.732124000	5.502647000	1.797724000
C	-4.917859000	6.867318000	1.634492000

C	-5.692912000	-0.907306000	1.557453000
C	-5.726480000	0.503953000	1.433303000
C	-5.941271000	-3.164660000	1.507406000
C	-6.099853000	2.714603000	1.067545000
C	-6.633022000	-1.959214000	1.397248000
C	-6.726410000	1.471576000	1.153015000
H	0.987434000	2.709302000	2.098483000
H	1.188058000	-2.646337000	2.097801000
H	1.402968000	-0.971605000	4.020582000
H	1.828114000	1.080904000	0.286763000
H	3.852485000	-0.867923000	4.370401000
H	4.281512000	1.185022000	0.608452000
H	-0.589383000	-4.633164000	2.038363000
H	-0.893966000	4.561645000	1.702348000
H	-1.918653000	-5.329343000	0.240256000
H	-2.093523000	-7.788679000	0.125626000
H	-2.130860000	5.033585000	-0.329175000
H	-2.437537000	7.466028000	-0.625296000
H	-3.509899000	-1.100023000	1.839663000
H	-3.550415000	0.842764000	1.581051000
H	-5.043830000	-5.229831000	3.177481000
H	-5.245655000	-7.688438000	3.058698000
H	-5.361387000	4.957063000	2.492995000
H	-5.694550000	7.383863000	2.192059000
H	-6.352973000	-4.157408000	1.400464000
H	-6.570519000	3.660170000	0.842434000
H	-7.689796000	-1.832048000	1.203037000
H	-7.780547000	1.264789000	1.024522000
N	6.806226000	0.279755000	2.874551000
N	-1.962928000	-1.590608000	1.843478000
N	-2.025482000	1.423324000	1.477020000
N	-3.870409000	-10.498606000	1.451962000
N	-4.447576000	10.147926000	0.431229000
N	-4.487105000	-1.494406000	1.801308000
N	-4.546851000	1.179807000	1.537263000

Isomer BC

C	0.017008000	2.557159000	1.831003000
C	0.046154000	0.003499000	1.941010000
C	0.196181000	-2.544238000	1.956689000
C	1.516026000	0.053634000	2.156300000
C	2.094587000	-0.507244000	3.310060000
C	2.379471000	0.656862000	1.223925000
C	3.465379000	-0.472625000	3.527937000
C	3.753039000	0.700799000	1.425634000

C	4.317466000	0.134415000	2.584807000
C	5.728675000	0.172246000	2.800152000
C	-0.522544000	-1.299692000	1.933667000
C	-0.597030000	1.258726000	1.770473000
C	-0.712501000	-3.582001000	1.898471000
C	-0.956476000	3.516978000	1.639407000
C	-2.019679000	-3.017485000	1.824496000
C	-2.204534000	2.849793000	1.469625000
C	-2.684243000	-5.876266000	0.832924000
C	-2.877126000	-7.246307000	0.724205000
C	-2.979299000	5.544091000	0.223233000
C	-3.229408000	6.887185000	-0.018653000
C	-3.306922000	-3.628222000	1.799530000
C	-3.450356000	-5.087963000	1.717887000
C	-3.529072000	3.363575000	1.359612000
C	-3.753545000	4.796862000	1.137682000
C	-3.853127000	-7.895778000	1.506747000
C	-4.067500000	-9.301136000	1.385700000
C	-4.275191000	7.551887000	0.653094000
C	-4.416183000	-5.757048000	2.502492000
C	-4.475819000	-2.815683000	1.812332000
C	-4.547688000	8.929110000	0.398551000
C	-4.618685000	-7.125446000	2.404455000
C	-4.640252000	2.475880000	1.443447000
C	-4.791127000	5.481696000	1.809424000
C	-5.051776000	6.823564000	1.576631000
C	-5.557283000	-0.909529000	1.693738000
C	-5.597890000	0.506082000	1.585394000
C	-5.863336000	-3.141915000	1.527346000
C	-6.047821000	2.674835000	1.141736000
C	-6.537881000	-1.941595000	1.457921000
C	-6.644569000	1.435201000	1.236467000
H	1.065320000	2.735022000	2.013918000
H	1.271103000	-2.632160000	1.992004000
H	1.443208000	-0.974942000	4.041463000
H	1.952865000	1.088924000	0.324360000
H	3.888196000	-0.904556000	4.430683000
H	4.401628000	1.161181000	0.685654000
H	-0.489707000	-4.638552000	1.913913000
H	-0.822097000	4.588682000	1.651490000
H	-1.953875000	-5.387438000	0.196802000
H	-2.190595000	5.039759000	-0.325236000
H	-2.289855000	-7.826312000	0.017394000
H	-2.631356000	7.432228000	-0.744101000
H	-2.755839000	-1.109737000	1.896099000
H	-2.794704000	0.901555000	1.628381000
H	-5.006913000	-5.175789000	3.202719000

H	-5.366409000	-7.613656000	3.023829000
H	-5.388022000	4.937428000	2.533507000
H	-5.853321000	7.325381000	2.112087000
H	-6.268632000	-4.130050000	1.356685000
H	-6.519255000	3.606206000	0.858750000
H	-7.588324000	-1.790914000	1.238486000
H	-7.686442000	1.192789000	1.063628000
N	6.880423000	0.201778000	2.978645000
N	-1.849370000	-1.643103000	1.842730000
N	-1.932328000	1.494019000	1.540458000
N	-4.243394000	-10.449543000	1.282391000
N	-4.350696000	-1.468720000	1.931168000
N	-4.424425000	1.163220000	1.715609000
N	-4.771455000	10.054770000	0.189815000

Isomer AB

C	0.087270000	-2.572379000	2.149861000
C	0.123485000	-0.010573000	1.965551000
C	1.585365000	0.043871000	2.161545000
C	2.207413000	-0.582417000	3.258984000
C	2.406791000	0.753340000	1.263712000
C	3.579252000	-0.515121000	3.451703000
C	3.779861000	0.832119000	1.443406000
C	4.386366000	0.194874000	2.542558000
C	5.794910000	0.274600000	2.737610000
C	-0.009007000	2.543833000	1.991831000
C	-0.494370000	-1.280804000	1.991988000
C	-0.576826000	1.218667000	1.804614000
C	-0.922441000	-3.510155000	2.105775000
C	-1.041937000	3.423793000	1.837180000
C	-2.167789000	-2.843405000	1.895354000
C	-2.236404000	2.632862000	1.580565000
C	-2.735697000	-5.599070000	0.735438000
C	-2.863441000	-6.965621000	0.544841000
C	-2.869155000	5.249984000	0.194149000
C	-3.009033000	6.595719000	-0.108523000
C	-3.451468000	-3.447320000	1.832541000
C	-3.546118000	3.191364000	1.470841000
C	-3.550291000	-4.900536000	1.652532000
C	-3.681658000	4.621567000	1.161347000
C	-3.822611000	-7.698723000	1.271615000
C	-3.963307000	-9.101045000	1.074801000
C	-3.980773000	7.372695000	0.552521000
C	-4.132256000	8.755992000	0.250440000
C	-4.507240000	-5.650790000	2.370353000

C	-4.644085000	-7.017692000	2.191452000
C	-4.650075000	5.414880000	1.812570000
C	-4.669231000	-2.716742000	1.885380000
C	-4.731217000	2.423864000	1.623411000
C	-4.802115000	6.761227000	1.519710000
C	-5.899234000	0.599931000	1.823833000
C	-5.927076000	-0.822062000	1.898201000
C	-5.970652000	-3.032703000	1.394070000
C	-6.066490000	2.722857000	1.114933000
C	-6.738874000	-1.867126000	1.402123000
C	-6.798705000	1.563813000	1.236914000
H	1.016182000	2.772147000	2.247024000
H	1.139878000	-2.763510000	2.288821000
H	1.588962000	-1.109455000	3.977937000
H	1.943084000	1.242526000	0.413462000
H	4.038085000	-0.993651000	4.312424000
H	4.397207000	1.377471000	0.734944000
H	-0.821304000	-4.576222000	2.239901000
H	-1.010225000	4.498607000	1.949951000
H	-2.011233000	-5.041217000	0.151791000
H	-2.130596000	4.652305000	-0.329395000
H	-2.237692000	-7.478827000	-0.180138000
H	-2.380175000	7.057790000	-0.864662000
H	-2.424571000	-0.684151000	1.567937000
H	-3.992058000	-0.794971000	2.572344000
H	-5.140835000	-5.135273000	3.084624000
H	-5.275622000	4.951581000	2.568256000
H	-5.379137000	-7.575552000	2.765259000
H	-5.546474000	7.357416000	2.040607000
H	-6.262585000	-4.000043000	1.012705000
H	-6.381795000	3.650766000	0.656555000
H	-7.747643000	-1.753477000	1.028667000
H	-7.818924000	1.389408000	0.915371000
N	6.947217000	0.342297000	2.898527000
N	-1.854928000	-1.489208000	1.836741000
N	-1.916091000	1.301659000	1.559735000
N	-4.077542000	-10.249949000	0.912055000
N	-4.257501000	9.889331000	0.007132000
N	-4.694353000	1.153167000	2.087389000
N	-4.733795000	-1.401218000	2.251932000

Isomer CD

C	0.118088000	-2.620396000	2.023755000
C	0.132554000	-0.059400000	1.901646000
C	1.589331000	-0.007098000	2.134589000

C	2.171447000	-0.652319000	3.243682000
C	2.443288000	0.717465000	1.280277000
C	3.532896000	-0.574445000	3.495864000
C	3.806896000	0.806376000	1.519486000
C	4.371077000	0.162267000	2.636950000
C	5.767573000	0.261456000	2.898826000
C	-0.009247000	2.499922000	1.928274000
C	-0.524258000	-1.322442000	1.899599000
C	-0.527585000	1.182299000	1.759546000
C	-0.886414000	-3.545873000	1.985693000
C	-1.046767000	3.395253000	1.783390000
C	-2.134175000	-2.811074000	1.833385000
C	-2.251587000	2.672260000	1.527534000
C	-2.797399000	-5.573922000	0.685550000
C	-2.865181000	5.322806000	0.207543000
C	-2.938212000	-6.942029000	0.509212000
C	-3.009812000	6.675349000	-0.055902000
C	-3.425285000	-3.418902000	1.832663000
C	-3.533785000	-4.877572000	1.665435000
C	-3.556367000	3.221582000	1.407755000
C	-3.697690000	4.654117000	1.132140000
C	-3.832698000	-7.672160000	1.316219000
C	-3.990948000	-9.075336000	1.131216000
C	-4.006819000	7.425097000	0.600172000
C	-4.159712000	8.815548000	0.339661000
C	-4.422604000	-5.624699000	2.466102000
C	-4.573283000	-6.993093000	2.302547000
C	-4.636157000	-2.683945000	1.934744000
C	-4.696485000	5.419604000	1.774028000
C	-4.743380000	2.449431000	1.533847000
C	-4.851178000	6.772610000	1.520727000
C	-5.865264000	-0.889940000	1.908838000
C	-5.933855000	0.528462000	1.792015000
C	-5.966574000	-3.092639000	1.490797000
C	-6.061798000	2.663061000	1.035642000
C	-6.737324000	-1.952395000	1.468366000
C	-6.789502000	1.481440000	1.195046000
H	1.015368000	2.730562000	2.176031000
H	1.178036000	-2.807867000	2.120797000
H	1.527810000	-1.203319000	3.921027000
H	2.014987000	1.211766000	0.414316000
H	3.959256000	-1.065921000	4.366014000
H	4.449006000	1.365342000	0.844500000
H	-0.790313000	-4.617595000	2.089394000
H	-0.999238000	4.467534000	1.898284000
H	-2.107563000	4.751250000	-0.317918000
H	-2.123973000	-5.013366000	0.045932000

H	-2.365348000	7.164712000	-0.781066000
H	-2.373697000	-7.458295000	-0.262261000
H	-2.434307000	0.490552000	1.353620000
H	-3.986272000	0.638893000	2.420809000
H	-4.993054000	-5.104692000	3.228729000
H	-5.258299000	-7.551126000	2.935119000
H	-5.343308000	4.929493000	2.494156000
H	-5.615793000	7.344019000	2.040045000
H	-6.254466000	-4.083165000	1.164603000
H	-6.395305000	3.571846000	0.556587000
H	-7.765983000	-1.856223000	1.140815000
H	-7.801672000	1.293963000	0.862581000
N	6.909713000	0.347210000	3.114931000
N	-1.873707000	-1.467041000	1.767638000
N	-1.881385000	1.332346000	1.514727000
N	-4.119880000	-10.223497000	0.976200000
N	-4.284359000	9.956023000	0.130125000
N	-4.641120000	-1.363856000	2.229855000
N	-4.751935000	1.180290000	2.042032000

Transition state (A-B)C‡

C	0.008692000	0.007950000	1.904246000
C	0.025845000	2.566576000	1.759212000
C	0.151212000	-2.518006000	1.970315000
C	1.479651000	0.038901000	2.124244000
C	2.042481000	-0.478831000	3.304955000
C	2.355150000	0.590401000	1.172391000
C	3.411973000	-0.450252000	3.531628000
C	3.728442000	0.625636000	1.381079000
C	4.277315000	0.104245000	2.568367000
C	5.686948000	0.136531000	2.794557000
C	-0.592547000	-1.281260000	1.917924000
C	-0.600944000	1.273811000	1.716871000
C	-0.740918000	-3.561837000	1.920153000
C	-0.945313000	3.531559000	1.578078000
C	-2.050420000	-2.996391000	1.821328000
C	-2.200988000	2.870342000	1.430550000
C	-2.656418000	-5.882517000	0.854068000
C	-2.827699000	-7.255737000	0.752253000
C	-3.003442000	5.578255000	0.224677000
C	-3.263106000	6.922783000	0.000765000
C	-3.322616000	-3.637737000	1.790611000
C	-3.449190000	-5.098222000	1.720060000
C	-3.526989000	3.383765000	1.347279000
C	-3.758440000	4.818827000	1.145351000

C	-3.808294000	-7.913993000	1.521643000
C	-4.001647000	-9.322609000	1.405700000
C	-4.298277000	7.577052000	0.698921000
C	-4.419722000	-5.776268000	2.491610000
C	-4.493617000	-2.832332000	1.790671000
C	-4.584355000	8.954336000	0.460355000
C	-4.600691000	-7.148199000	2.399853000
C	-4.633620000	2.491667000	1.440605000
C	-4.784111000	5.493839000	1.844741000
C	-5.053342000	6.837224000	1.630998000
C	-5.552632000	-0.891231000	1.670564000
C	-5.589768000	0.522187000	1.574254000
C	-5.885890000	-3.127868000	1.559709000
C	-6.045259000	2.693719000	1.164065000
C	-6.542908000	-1.912650000	1.490477000
C	-6.642465000	1.453901000	1.254668000
H	1.078680000	2.736929000	1.923244000
H	1.226932000	-2.592405000	2.018315000
H	1.379670000	-0.907599000	4.049846000
H	1.938451000	0.990448000	0.253445000
H	3.824038000	-0.846140000	4.455555000
H	4.387777000	1.045831000	0.626860000
H	-0.511661000	-4.616770000	1.958103000
H	-0.804200000	4.602438000	1.582958000
H	-1.921321000	-5.388025000	0.228081000
H	-2.218359000	-7.832052000	0.061246000
H	-2.223685000	5.082403000	-0.343933000
H	-2.681596000	7.476924000	-0.731198000
H	-2.775697000	0.907497000	1.591581000
H	-3.047628000	-1.173439000	1.860738000
H	-5.028484000	-5.200771000	3.180979000
H	-5.351506000	-7.642908000	3.010240000
H	-5.364673000	4.940584000	2.575153000
H	-5.846435000	7.330645000	2.186362000
H	-6.316326000	-4.110613000	1.427588000
H	-6.519937000	3.628213000	0.897779000
H	-7.598491000	-1.747745000	1.312727000
H	-7.686918000	1.213895000	1.094855000
N	6.837147000	0.162405000	2.982820000
N	-1.924070000	-1.616116000	1.825464000
N	-1.932495000	1.516410000	1.500321000
N	-4.161539000	-10.473754000	1.306738000
N	-4.347081000	-1.479974000	1.872331000
N	-4.414631000	1.174091000	1.695192000
N	-4.821540000	10.079333000	0.262717000

Transition state (B-C)A‡

C	0.087280000	-2.529936000	2.196145000
C	0.191947000	0.017615000	2.094282000
C	1.648414000	0.061247000	2.281281000
C	2.262438000	-0.599606000	3.365171000
C	2.482838000	0.773317000	1.396118000
C	3.634135000	-0.554346000	3.560740000
C	3.856624000	0.826199000	1.577683000
C	4.453270000	0.162844000	2.667141000
C	5.862066000	0.219583000	2.865371000
C	-0.039117000	2.562669000	1.884701000
C	-0.481582000	-1.221936000	2.132922000
C	-0.528691000	1.215899000	1.882201000
C	-0.948948000	-3.419547000	2.092496000
C	-1.128020000	3.367467000	1.681669000
C	-2.176236000	-2.680916000	1.966933000
C	-2.298427000	2.527873000	1.586685000
C	-2.752024000	-5.314890000	0.527183000
C	-2.836903000	-6.657447000	0.194650000
C	-3.037331000	4.979067000	-0.073669000
C	-3.200179000	6.284431000	-0.511623000
C	-3.440789000	-3.336038000	1.919424000
C	-3.491988000	-4.770379000	1.597666000
C	-3.607351000	3.082137000	1.482323000
C	-3.677194000	-7.518352000	0.928396000
C	-3.745117000	4.479707000	1.038888000
C	-3.772948000	-8.897463000	0.588369000
C	-4.087959000	7.149775000	0.157893000
C	-4.258124000	8.494652000	-0.278728000
C	-4.329017000	-5.645827000	2.320823000
C	-4.423593000	-6.991263000	2.000282000
C	-4.628664000	5.359506000	1.697560000
C	-4.681088000	-2.689040000	2.123370000
C	-4.793525000	2.366190000	1.770552000
C	-4.801376000	6.667800000	1.272205000
C	-5.958717000	0.578714000	2.181393000
C	-5.973570000	-0.838718000	2.299212000
C	-6.022361000	-3.046075000	1.784758000
C	-6.167712000	2.668798000	1.388538000
C	-6.815089000	-1.903324000	1.897281000
C	-6.900705000	1.532528000	1.651613000
H	0.983524000	2.865691000	2.053989000
H	1.139063000	-2.753224000	2.292874000
H	1.635284000	-1.136147000	4.069508000
H	2.031751000	1.275548000	0.546816000
H	4.084973000	-1.057513000	4.411711000

H	4.482981000	1.371145000	0.876700000
H	-0.887984000	-4.496371000	2.137078000
H	-1.144009000	4.447057000	1.656315000
H	-2.117423000	-4.654239000	-0.053929000
H	-2.251111000	-0.116429000	1.804295000
H	-2.268381000	-7.052810000	-0.642505000
H	-2.362677000	4.312340000	-0.600291000
H	-2.653737000	6.646336000	-1.378203000
H	-3.950737000	-0.766825000	2.645971000
H	-4.900477000	-5.248791000	3.153487000
H	-5.065402000	-7.650547000	2.578477000
H	-5.172912000	4.995774000	2.562707000
H	-5.478264000	7.333499000	1.800739000
H	-6.334037000	-4.019350000	1.435691000
H	-6.519521000	3.580976000	0.925632000
H	-7.865072000	-1.815967000	1.651859000
H	-7.953145000	1.365689000	1.453791000
N	7.015278000	0.266958000	3.028872000
N	-1.856500000	-1.318808000	1.996860000
N	-1.893580000	1.205605000	1.710930000
N	-3.849146000	-10.026783000	0.308818000
N	-4.394315000	9.598248000	-0.628987000
N	-4.723915000	1.121287000	2.285838000
N	-4.738501000	-1.387949000	2.520689000

Transition state (A-B)D‡

C	0.152072000	-2.562714000	2.016208000
C	1.449918000	0.039835000	2.146901000
C	2.022145000	-0.472771000	3.325738000
C	2.319569000	0.596444000	1.192008000
C	3.392261000	-0.431150000	3.549496000
C	3.693046000	0.645122000	1.397622000
C	4.250070000	0.131823000	2.584899000
C	5.659499000	0.181671000	2.810289000
C	-0.016385000	2.515163000	1.789663000
C	-0.022806000	-0.017775000	1.933633000
C	-0.598492000	-1.316854000	1.946781000
C	-0.680914000	1.228677000	1.751543000
C	-0.758036000	-3.583024000	1.954986000
C	-0.961015000	3.488447000	1.586822000
C	-2.064263000	-2.969024000	1.839101000
C	-2.222121000	2.820557000	1.438539000
C	-2.636809000	-5.833906000	0.807790000
C	-2.772040000	-7.208836000	0.682439000
C	-2.932625000	5.530739000	0.177965000

C	-3.140500000	6.879211000	-0.072137000
C	-3.331686000	-3.627001000	1.802556000
C	-3.438349000	-5.087325000	1.699111000
C	-3.527175000	3.380099000	1.335546000
C	-3.719554000	4.816190000	1.109016000
C	-3.726907000	-7.906693000	1.449443000
C	-3.877023000	-9.319129000	1.317165000
C	-4.156859000	7.583539000	0.604639000
C	-4.387852000	-5.803118000	2.461859000
C	-4.390830000	8.964973000	0.336080000
C	-4.527645000	-2.857767000	1.821412000
C	-4.533385000	-7.177527000	2.346257000
C	-4.651537000	2.515145000	1.441567000
C	-4.723686000	5.541748000	1.788610000
C	-4.942590000	6.889818000	1.546952000
C	-5.626006000	0.521495000	1.607497000
C	-5.626499000	-0.889031000	1.718101000
C	-5.909679000	-3.138808000	1.585480000
C	-6.059451000	2.712724000	1.253577000
C	-6.585722000	-1.922212000	1.529050000
C	-6.662570000	1.469425000	1.363687000
H	1.036837000	2.668526000	1.970079000
H	1.226275000	-2.653727000	2.087466000
H	1.366187000	-0.907905000	4.072911000
H	1.897850000	0.990974000	0.273023000
H	3.810310000	-0.823183000	4.472506000
H	4.346700000	1.071136000	0.641676000
H	-0.554773000	-4.643529000	2.003158000
H	-0.809349000	4.558365000	1.585528000
H	-1.919137000	-5.307678000	0.187717000
H	-2.152539000	-7.756317000	-0.022763000
H	-2.168063000	4.995620000	-0.375087000
H	-2.533168000	7.397577000	-0.809238000
H	-3.202779000	0.950749000	1.639436000
H	-3.466622000	-1.095533000	1.944811000
H	-5.004732000	-5.257084000	3.168119000
H	-5.264036000	-7.703487000	2.954980000
H	-5.324691000	5.027888000	2.531143000
H	-5.718312000	7.423159000	2.089890000
H	-6.334483000	-4.121563000	1.442265000
H	-6.545954000	3.653180000	1.039125000
H	-7.641668000	-1.773624000	1.346021000
H	-7.717380000	1.241762000	1.275216000
N	6.809091000	0.223412000	2.999516000
N	-1.925187000	-1.599887000	1.841493000
N	-2.019737000	1.459325000	1.543045000
N	-3.999992000	-10.473845000	1.206636000

N	-4.433775000	-1.500287000	1.933397000
N	-4.434209000	1.181074000	1.660787000
N	-4.583556000	10.094037000	0.114883000

Transition state (A-D)B‡

C	0.140479000	-2.569031000	2.089037000
C	0.159590000	-0.017018000	1.903192000
C	1.614587000	0.033093000	2.142312000
C	2.191164000	-0.587925000	3.268749000
C	2.479805000	0.713279000	1.262226000
C	3.557525000	-0.545781000	3.502205000
C	3.848562000	0.766196000	1.482508000
C	4.408944000	0.132074000	2.608074000
C	5.813922000	0.177131000	2.839613000
C	-0.002978000	2.548117000	1.786285000
C	-0.501126000	1.212356000	1.688602000
C	-0.506289000	-1.276520000	1.935049000
C	-0.853535000	-3.503273000	2.041378000
C	-1.059021000	3.414040000	1.603621000
C	-2.101468000	-2.781787000	1.842481000
C	-2.250498000	2.648454000	1.390933000
C	-2.721682000	-5.629791000	0.820694000
C	-2.823495000	-7.012309000	0.765828000
C	-3.037442000	5.328279000	0.147715000
C	-3.232273000	6.692521000	-0.011333000
C	-3.379364000	-3.398522000	1.785763000
C	-3.460769000	-4.872752000	1.750780000
C	-3.571425000	3.131823000	1.256739000
C	-3.679919000	-7.694754000	1.651415000
C	-3.762837000	4.585069000	1.100315000
C	-3.794772000	-9.114299000	1.594683000
C	-4.170580000	7.372536000	0.789634000
C	-4.312905000	-5.570659000	2.629213000
C	-4.376801000	8.773745000	0.634166000
C	-4.423849000	-6.953002000	2.588887000
C	-4.605895000	-2.688420000	1.725614000
C	-4.696660000	5.281123000	1.894600000
C	-4.732900000	2.312918000	1.276072000
C	-4.900905000	6.644862000	1.749345000
C	-5.927157000	-3.189400000	1.412472000
C	-5.963128000	-0.951030000	1.523229000
C	-5.994834000	0.471369000	1.367081000
C	-6.088013000	2.675074000	0.953724000
C	-6.772172000	-2.096653000	1.285587000
C	-6.868827000	1.525631000	1.016024000

H	1.020255000	2.809840000	2.008578000
H	1.199735000	-2.746510000	2.206995000
H	1.540393000	-1.099481000	3.969888000
H	2.057405000	1.191757000	0.384533000
H	3.979195000	-1.024503000	4.381767000
H	4.498587000	1.284140000	0.782761000
H	-0.748386000	-4.573128000	2.151639000
H	-1.028707000	4.491633000	1.654670000
H	-2.072257000	-5.107587000	0.125946000
H	-2.254387000	-7.577109000	0.032674000
H	-2.323413000	4.808753000	-0.482994000
H	-2.412266000	0.480036000	1.388820000
H	-2.672576000	7.243592000	-0.762015000
H	-4.170046000	-0.098084000	1.772637000
H	-4.886837000	-5.003398000	3.354779000
H	-5.078404000	-7.472916000	3.283081000
H	-5.256384000	4.727394000	2.640942000
H	-5.616115000	7.163434000	2.382032000
H	-6.184895000	-4.227986000	1.258232000
H	-6.417502000	3.666020000	0.675829000
H	-7.823607000	-2.107765000	1.026307000
H	-7.929828000	1.444649000	0.816897000
N	6.963552000	0.214589000	3.029303000
N	-1.851207000	1.325789000	1.438522000
N	-1.853463000	-1.432694000	1.779573000
N	-3.886960000	-10.275393000	1.544600000
N	-4.544034000	9.920822000	0.510174000
N	-4.692669000	-1.337645000	1.819071000
N	-4.729733000	0.972288000	1.526142000
